# S100A1 blocks the interaction between p53 and mdm2 and decreases cell proliferation activity

**DOI:** 10.1371/journal.pone.0234152

**Published:** 2020-06-04

**Authors:** Deepu Dowarha, Ruey-Hwang Chou, Chin Yu

**Affiliations:** 1 Department of Chemistry, National Tsing Hua University, Hsinchu, Taiwan; 2 Graduate Institute of Biomedical Sciences and Center for Molecular Medicine, China Medical University, Taichung, Taiwan; 3 Department of Biotechnology, Asia University, Wufeng, Taichung, Taiwan; Russian Academy of Medical Sciences, RUSSIAN FEDERATION

## Abstract

About 50% of human cancers across the globe arise due to a mutation in the p53 gene which gives rise to its functional inactive form, and in the rest of the cancer the efficacy of active p53 (wild-type) is hindered by MDM2-mediated degradation. Breakdown of the p53-MDM2 association may constitute an effective strategy to stimulate or reinstate the activity of wild type p53, thereby reviving the p53 tumor suppressor capability. S100A1 has been revealed to associate with the N-terminal domain of MDM2 and p53 protein. We utilized NMR spectroscopy to study the interface amongst the S100A1 and N-terminal domain of MDM2. Additionally, the S100A1-MDM2 complex generated through the HADDOCK program was then superimposed with the p53 (peptide) -MDM2 complex reported earlier. The overlay indicated that a segment of S100A1 could block the interaction of p53 (peptide) -MDM2 complex significantly. To further justify our assumption, we performed HSQC-NMR titration for the S100A1 and p53 N-terminal domain (p53-TAD). The data obtained indicated that the S100A1 segment comprising nearly 17 residues have some common residues that interact with both MDM2 and p53-TAD. Further, we synthesized the 17-residue peptide derived from the S100A1 protein and attached it to the cell-penetrating HIV-TAT peptide. The HSQC-NMR competitive binding experiment revealed that Peptide 1 could successfully interfere with the p53-MDM2 interaction. Furthermore, functional effects of the peptide was validated in cancer cells. The results showed that Peptide 1 effectively inhibited cell proliferation, and increased the protein levels of p53 and its downstream p21 in MCF-7 cells. Treatment of Peptide 1 resulted in cell cycle arrest at G2/M phase, and also induced apoptotic cell death at higher concentration. Taken together, the results suggest that disruption of the interaction of p53 and MDM2 by Peptide 1 could activate normal p53 functions, leading to cell cycle arrest and apoptotic cell death in cancer cells. We proposed here that S100A1 could influence the p53-MDM2 interaction credibly and possibly reactivates the wild type p53 pathway.

## Introduction

The p53 protein is widely known as the “guardian of the genome” pertaining to its ability to inhibit tumor development. [1] In response to numerous cellular stressors, p53 acts as a tumor suppressor by precisely controlling its transcription activity. It regulates the expression of downstream specific genes whose protein output plays role in cell growth arrest, senescence, or programmed cell death, thereby prohibiting oncogenesis. [2]

The p53 protein is active in its tetrameric form, and each identical chain has 393 residues. [3] The full-length p53 protein consists of an N- terminal domain (NTD, 1–93), the intermediate DNA-binding domain (DBD, 102–292), and the C-terminal domain (CTD, 293–393). [4, 5]

The p53 N-terminal domain (NTD, 1–93) is natively unfolded, lacking tertiary structure and highly deficient in secondary structure elements. [5] The p53 NTD domain is rich in proline residues (62–91) and contains two TAD sub-domains: TAD1 (1–40) and TAD2 (43–73). [6, 7] Much of the tumor suppressor capability of p53 relies upon the region of TAD1, and TAD2 sub-domains and mutations in these regions lead to the loss of tumor suppression activity of p53 protein. [8]

Functional inactivation of p53 induced by mutations or deletions accounts for near about 50% of human cancers around the world [9], mostly seen in cancers related to the breast, prostate, and cancers in which these mutations are connected with poor prognosis and enhanced chemo reluctance. [10, 11] Since p53 plays a pivotal role in regulating several cellular processes, its activity level is severely monitored.

The MDM2 oncogene is a principal cellular controller and suppressor of p53. [12, 13] The MDM2 gene encodes a full-length protein of 491 amino acids [14] and is expressed as different isoforms. [15, 16] Sequence studies on MDM2 indicated another MDM2 family member, identified as MDMX. [17] MDM2 and MDMX are structurally related protein and shows greatest similarity in their N-terminal domain which binds to a short α-helical stretch within the p53 N-terminal domain. [18–20] The MDM2 residues including L54, L57, I61, M62, Y67, Q72, V75, F86, F91, V93, I99, Y100, and I103 are particularly important for p53 interactions. Out of these 13 residues, 10 residues are conserved in MDMX. [21] However, MDM2 acts as an E3 ubiquitin ligase that targets p53 for ubiquitination and degradation, and MDMX lacks this function. [22, 23] The N-terminal domain of MDM2 residues close to 100, interacts with the p53-TAD region and interrupts the p53 transcription activity. [24, 25]

In normal cells, the level of p53 activity is meticulously monitored by MDM2, and the p53-MDM2 interaction forms a negative feedback loop or self-regulating response loop that confines the growth suppressing action of p53. [26] MDM2 and p53 modulate each other by mutually employing the auto-regulatory feedback loop. [27, 28]

Cells harboring wild-type p53, upon activation by numerous stimuli, transcribe the MDM2 gene and result in the elevated level of MDM2 mRNA and protein. The MDM2 protein expressed then associates directly with the p53 protein, mediated via the N-terminal domain, and inhibits the p53 activity. [25] This MDM2 mediated p53 inactivation could be explained through three possible mechanisms: (i) MDM2 binding with p53 forming MDM2-p53 complex inhibits the p53 activity as an effective transcription factor towards its targeted DNA molecule; [29, 30] (ii) MDM2 induces stepwise proteasomal degradation of p53 by directly ubiquitinating p53 by its E3 ligase activity, [31–33] and (iii) MDM2 reduces the transcriptional ability of p53 by facilitating nuclear export of p53. [34] Utilizing one of these three possible routes, MDM2 performs as a negative controller of p53 in cells consisting of wild-type p53. As a controller of the p53 tumor suppressor role, MDM2, once expressed in excess amount, is tumorigenic in nature. [35, 36]

As MDM2 primarily work as a key manager of p53 tumor suppressor activity, chemical moieties targeting MDM2 function could reactivate the action of wild-type p53 progressively. [37–39] Therefore, custom-designed small molecules such as peptides targeting disruption of MDM2-p53 protein-protein association could facilitate upsurge in p53 protein and switch on the p53 transcriptional activity. Such peptides capable of reviving the p53 activity may have a strong therapeutic potential in malignant tumors retaining wild type p53. [40–43]

S100A1 belongs to the human S100 family of proteins consisting of 21 members, typically ranging from 10–12 kDa in size. With a helix–loop–helix pattern, these proteins are site-specific and form homo and heterodimers. [44–46] These proteins interact with copper, zinc, and calcium ions, and calcium-binding facilitates a variety of extracellular and intracellular governing consequences of these proteins. [47, 48] S100A1 protein was found to associate with the p53 peptide (residues, 367–393) derived from the C-terminal domain. Further, it was also found to connect with the p53 tetramerization domain (residues, 325–355) and influencing its oligomerization state. The S100A1 protein only interacted with the TET domain when its oligomerization state was lowered (monomers or dimers). [49] Additionally, S100A1 could bind to the monomeric fragment of the C-terminal domain (residues, 293–393) but unable to form a complex with the tetrameric form. Moreover, fluorescence anisotropy experiments showed the binding of S100A1 to the p53 transactivation domain (residues, 1–57). [50] Interestingly, S100A1 could bind to the N-terminus and C-terminus of the p53 protein, which is also the main site for the posttranslational modifications (PTMs) and comprises target sites for interacting partners. The influential role of the single-site phosphorylation in the p53 N-terminus towards its binding to S100A1 was carried out by the fluorescence anisotropy experiment. The data output indicated that upon phosphorylation, p53 N-terminus showed an increased affinity for S100A1 implying the stimulating effect in the p53 activation. [51] Besides this, the S100A1 was also found to associate with the MDM2 N- terminal domain (2–125) and does not lead to the formation of a ternary complex with MDM2 and p53. [52] As S100A1 has shown to interact with the N-terminal domains of the MDM2 and p53 protein, it was of interest to analyze the binding residues of S100A1 in MDM2 and p53 protein. We engaged heteronuclear single-quantum correlation-NMR (HSQC-NMR) spectroscopy [53, 54] to define the interfaces flanked by the S100A1 and MDM2 N-terminal domain. Further, the S100A1-MDM2 complex generated through the HADDOCK (High Ambiguity Driven protein-protein Docking) program [55–57] was then superimposed with the p53 (peptide)-MDM2 complex, which was already reported. The overlay of the 3D structures of the S100A1-MDM2 and p53 (peptide)-MDM2 complex indicated that a segment of S100A1 could significantly obstruct the interaction between p53 (peptide) and MDM2. To further justify our assumption, we performed HSQC-NMR titration for the S100A1 and p53 N-terminal domain. The data obtained indicated that the S100A1 segment comprising nearly 17 residues have some common residues interacting with both MDM2 and p53. Further, we constructed the 17 residue peptide derived from the S100A1 protein and attached it to the cell-penetrating HIV-TAT (Human immunodeficiency virus- Transcription- transactivating) peptide. [58, 59] We then used this fusion peptide, Peptide 1, for performing the HSQC-NMR competitive binding experiment, followed by the water-soluble tetrazolium-1 (WST-1) assay in the MCF-7 and AGS cancer cell line. Our study showed that the Peptide 1 could successfully interfere with the p53-MDM2 interaction thereby potentially lowering the viable cell count by possible means of disrupting the p53-MDM2 interaction and probably reactivating the wild-type p53 pathway. We propose here that the S100A1-derived peptide (Peptide 1) can obstruct the p53-MDM2 interaction and possibly reactivates the wild-type p53 pathway.

## Materials and methods

### Materials

All protein-expressing bacterial cells were grown in M9 media and isotope-labeled (^15^N- ammonium chloride) samples having 10% deuterium oxide (D_2_O) were utilized for HSQC- NMR experiments. The Milli-Q water was utilized for the development of entire buffers and were additionally purified with a 0.22-μm filter instrument. All purified proteins were identified by the sodium dodecyl sulfate (SDS)—polyacrylamide gel electrophoresis (PAGE) and high-performance liquid chromatography (HPLC) techniques, and confirmation was done through electrospray ionization mass spectrometry (ESI-MS) analysis. The MCF7 breast cancer cell line was procured from the American Type Culture Collection (ATCC, HTB-22).

### Purification of p53-TAD

The plasmid expressing p53-TAD (residues, 1–73) was a kind gift from Prof. Gary Daughdrill (University of South Florida). The p53-TAD protein was expressed and purified as mentioned earlier. [4]

### Purification of MDM2 (17–125)

In this paper, MDM2 (17–125) and/or MDM2 are used interchangeably unless otherwise indicated, meaning the N-terminal domain of MDM2 containing residues 17–125. The plasmid expressing human MDM2 protein was acquired from Prof. Gary Daughdrill (Addgene plasmid # 62063; http://n2t.net/addgene: 62063; RRID: Addgene_62063) and was expressed and purified as mentioned earlier. [60]

### Purification of S100A1

S100A1 protein was produced in BL21 (DE3) E. coli system utilizing a pET20b + expression vector system consisting of human S100A1 cDNA. It was purified as per the protocol published earlier. [61]

### Peptide synthesis

Peptide 1 (HIV-TAT + S100A1 derived peptide; Y-G-R-K-K-R-R-Q-R-R-R-V-V-L-V-A-A-L-T-V-A-C-N-N-F-F-W-E) and Peptide 2 (HIV-TAT + Scramble of S100A1 derived peptide; Y-G-R-K-K-R-R-Q-R-R-R-V-A-T-W-N-F-E-V-L-N-A-V-C-F-A-L-V) with ≥ 95% purity were purchased from Kelowna International Scientific Inc.

### HSQC- NMR titration experiments

All the experiments pertaining to HSQC-NMR titrations were performed by utilizing a cryogenic probe inside a Varian 700-MHz spectrometer running at 25°C. By means of an NMR buffer (20 mM Tris-HCl, 15 mM NaCl, 20 mM CaCl_2_, 20 mM DTT, 0.1% NaN_3_, 10% D_2_O, 7.2 pH), unlabeled S100A1 protein was added to ^15^N-labeled MDM2 at a molar ratios of 0:1, 0.2:1, 0.4:1, 0.6:1, 0.8:1, and 1:1. The converse titration studies (^15^N-labeled S100A1 and unlabeled MDM2) with a similar procedure and set-up were also performed, respectively.

Another set of HSQC-NMR experiments, comprising unlabeled p53-TAD and ^15^N-labeled S100A1 (NMR buffer, 20 mM Tris-HCl, 15 mM NaCl, 20 mM CaCl_2_, 20 mM DTT, 0.1% NaN_3_, and 10% D_2_O; pH 7.2) along with converse set-up, i.e., ^15^N-labeled p53-TAD and unlabeled S100A1 (NMR buffer, 25 mM Tris-HCl, 5 mM CaCl_2_, 114 mM NaCl, 0.05 mM EDTA, 1 mM DTT, and 10% D_2_O; pH 7.2) were similarly carried out as mentioned above at molar ratios of 0:1, 0.5:1, 0.75:1, and 1:1. The generated NMR spectra were processed through the VNMRJ 2.3 program and additionally analyzed by Sparky 3.1 software [62] for the identification of significant variations in the peak intensities, any associated chemical perturbations, or shifts in the cross-peaks of the protein.

### Molecular docking

The complex model of S100A1-MDM2 was generated by employing the HADDOCK program (version 2.2). Molecular docking tests were carried out by using NMR coordinates of S100A1 and MDM2, which were retrieved from the Protein Data Bank (PDB ID: 2LP3 and PDB ID: 1YCR, respectively). HSQC-NMR titration data analysis resulted in the number of residues showing significant chemical shift perturbation or low intensity. These identified residues were loaded in the AIR (Ambiguous Interaction Constraints) constraints and were grouped into passive and active residues based on the NACCESS program. [63] Residues showing a relatively available surface region > 40 percentages, in addition to backbone and side chains, were mapped as active, while those < 40 percentages were mapped as passive residues. Overall, 2000 assemblies were assigned for rigid-body docking by means of the HADDOCK program, which also included the optimized potential for liquid simulation parameters. [64] One thousand models were then set for semi-flexible refinement, and finally, 200 models with the lowest energy were scrutinized. In the last stage, PyMOL software [65] was utilized to study the all-possible structures generated in clusters.

### HSQC- NMR competitive binding experiment

In the first experiment, unlabeled Peptide 1 was added to the ^15^N-labeled MDM2 in a 1:1 ratio.

In the second experiment, unlabeled p53-TAD was added to the ^15^N-labeled MDM2 in a 1:1 ratio. Later, in continuation of the second experiment, unlabeled Peptide 1 was added in a 1:1 ratio. All the experiments were carried out in the NMR buffer containing 20 mM Tris-HCl, 15 mM NaCl, 20 mM CaCl_2_, 20 mM DTT, 0.1% NaN_3_, and 10% D_2_O; pH 7.2.

### Fluorescence experiment

In our fluorescence experiment, we have used the N-terminal domain of p53 (1–73) and MDM2 (17–125). The p53 (1–73) contains two tryptophan residues (W23 and W53) and MDM2 (17–125) does not contain any tryptophan residues. The tyrosine and phenylalanine residues of MDM2 will not interfere with the excitation wavelength (295 nm) in this situation. A Hitachi F-2500 fluorescence spectrophotometer instrument was utilized to measure the emission spectrum of the tryptophan fluorescence of p53 protein. The p53 tryptophan was excited at a wavelength of 295 nm, and the successive variations in the emission spectra were observed by scanning from 305 to 450 nm with a slit width of 5 nm. We first studied the interaction among p53 (1–73) and MDM2 (17–125). MDM2 (17–125) was added to the p53 (1–73), which was having a concentration of 4 μM and the buffer consists of 25 mM Tris-HCl, 5 mM CaCl_2_, 114 mM NaCl, 0.05 mM EDTA, and 1 mM DTT; pH 7.2. MDM2 concentration was increased (0 μM to 12.8 μM) and the data were plotted as the total concentration of the MDM2 against the relative intensity. The K_d_ was calculated from the following equation [66] by plotting the nonlinear curve using the GraphPad Prism 6 software.

$$f=\frac{({\left[P\right]}_{T}+{\left[L\right]}_{T}+[K_{d}])-\sqrt{{\left({\left[P\right]}_{T}+{\left[L\right]}_{T}+[K_{d}]\right)}^{2}-4{\left[P\right]}_{T}{\left[L\right]}_{T}}}{2{\left[P\right]}_{T}}$$ (Eq 1)

Where, f represents the fractional change, K_d_ is the dissociation constant, and [P]_T_ and [L]_T_ corresponds to the total concentration of p53 and MDM2, respectively.

In the second case, Peptide 1 was added in the increasing concentration (0 μM to 12.0 μM) to the p53, which was having a concentration of 2 μM and the buffer consists of 20 mM Tris-HCl, 15 mM NaCl, 20 mM CaCl_2_, 20 mM DTT, and 0.1% NaN_3_; pH 7.2. We used the same method as indicated earlier and calculated the dissociation constant amongst Peptide 1 and p53 (1–73).

### Circular Dichroism (CD) spectroscopy

CD experiments were carried out with an Aviv Model 410 CD spectrometer. Far-UV CD spectra were obtained at 25°C in Phosphate buffer (with Ca^2+^ and Mg^2+^ ions) having pH 7.4, using a 1 mm path length quartz cuvette. The concentration of S100A1 protein and Peptide 1 was kept at around 50 μM for far-UV CD experiments.

### Cell proliferation assay

The WST-1 [4-[3-(4-iodophenyl)-2-(4-nitrophenyl)-2H-5-tetrazolio]-1, 3-benzene disulfonate] cell proliferation assay was carried out as per the manufacturer’s (Roche) guidelines.

### Western blotting

Cells were harvested and washed twice with phosphate buffer saline (PBS, containing 137 mM NaCl, 2.7 mM KCl, 10 mM Na_2_HPO_4_, 2 mM KH_2_PO_4_). The total cell lysate was extracted by sonication in RIPA Buffer (50 mM Tris at pH 7.5, 150 mM NaCl, 1 mM EDTA, 0.25% Na-deoxycholate, 1% NP-40, 1 mM NaF, 1 mM Na_3_VO_4_, 1 mM PMSF, 1 μg/ml aprotinin). The soluble extraction was collected from the supernatant after centrifugation at 15000 g for 10 min. Cell lysate was separated by sodium dodecyl sulfate polyacrylamide gel electrophoresis (SDS-PAGE) and transferred to a PVDF membrane. The membrane was then blocked with 5% skim milk in PBST buffer (PBS containing 0.1% Tween-20) at room temperature for 1h, and hybridized with primary antibody with gentle agitation at 4°C overnight. After washing with PBST, the membrane was incubated with HRP-conjugated secondary antibody (Chemicon, MA, USA) at room temperature for 1h. The immunoreactive band was visualized by the enhanced chemiluminescence (ECL) detection reagent (GE Healthcare, Amersham Place, UK).

### Cell cycle analysis

Cells were seeded at the density of 1x 10^6^ cells/dish the day before experiment, and then treated with indicated concentrations of peptide 1 or peptide 2 for 48h. After treatment, cells were collected and fixed with 70% ethanol at 4°C for 1h. The fixed cells were washed with PBS, and then treated with 100 μg/ml RNAse A and stained with 10 μg/ml propidium iodide (PI) for 30 min in dark. Subsequently, the PI stained cells were applied to a flow cytometer (BD FACS Verse) for cell cycle analysis. The distribution of cell cycle was analyzed by Flowjo software (BD Biosciences).

## Results and discussion

We studied the interaction of S100A1 protein to the N- terminal domain of MDM2 (residues, 17–125) and p53-TAD (residues, 1–73) by employing the NMR experiments. The ^1^H−^15^N HSQC-NMR methods are well established to identify the binding interface between proteins or amid proteins and their connected ligands.

### Interaction amongst labeled S100A1 and unlabeled MDM2

The affected residues in the S100A1 protein upon interaction with MDM2 could be observed well by examining the HSQC-NMR spectra of free S100A1 and compared to those of the S100A1−MDM2 complex.

**Fig 1:**
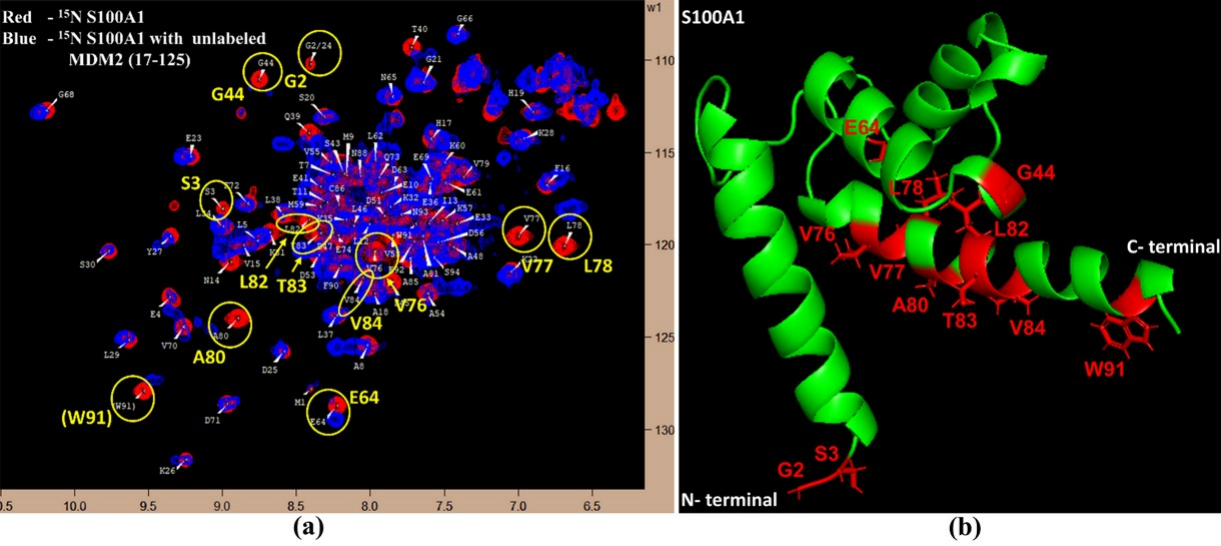
Exploration of the connected interface (S100A1/MDM2) section in S100A1. (a) The ^1^H−^15^N HSQC spectra overlay of free ^15^N S100A1 (red) and in complex with unlabelled MDM2 (blue). Cross-peaks displaying variation in their magnitude are highlighted in yellow. Residues were assigned as published earlier. [61] (b) A picture illustration of the S100A1 protein with residues demonstrating a change in cross-peak signals portrayed by the red color.

Fig 1A demonstrates the overlapped HSQC-NMR spectra of free nitrogen-15-labeled S100A1 (red color) and nitrogen-15-labeled S100A1 complexed with unlabelled MDM2 (blue color). Upon the introduction of unlabelled MDM2 to the nitrogen-15-labeled S100A1, some of the NMR signals exhibit a reduction in their intensity. The detected HSQC-NMR signs of the S100A1−MDM2 complex formation were considerably lower to those attained with free S100A1. This manifestation was due to ^15^N nuclei being closer to the interface section of the proteins flanked by S100A1 and MDM2. The ^15^N nuclei in the vicinity of the interacting areas were influenced by the existence of other proteins in a close locality, which reduced the cross-peaks on the HSQC NMR spectrum. The complex formation of S100 proteins with their target proteins noticeably affected the protein residues.

The ^1^H−^15^N HSQC-NMR spectra obtained for the free S100A1 and in complex with unlabelled MDM2 were overlapped to categorize the S100A1 residues adjacent to the interface area undergoing perturbation or decrease in the intensity. These affected residues included G2, S3, G44, E64, V76, V77, L78, A80, L82, T83, V84, and W91, and all are highlighted in Fig 1A and are also labeled in the S100A1 cartoon (Fig 1B).

### Interaction amongst unlabeled S100A1 and labeled MDM2

**Fig 2:**
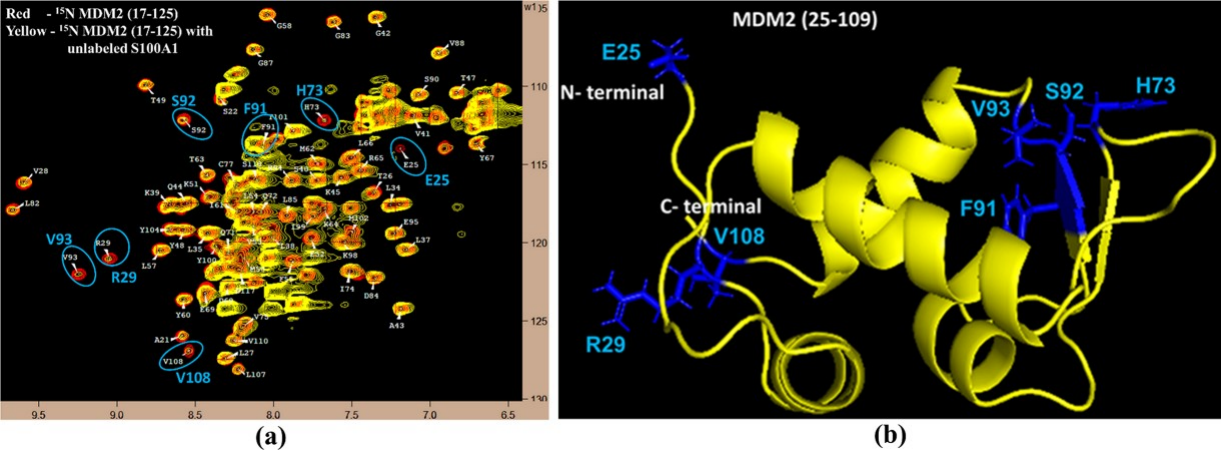
Exploration of the connected interface (S100A1/MDM2) section in MDM2. (a) The ^1^H−^15^N HSQC spectra overlay of free ^15^N MDM2 (red) and in complex with unlabelled S100A1 (yellow). Cross-peaks illustrating variation in their magnitude is highlighted in blue. Residues were assigned as published earlier. [67] (b) A picture showing the MDM2 protein with residues displaying change in cross-peak signs, which are characterized by the blue color.

The HSQC-NMR experiments were performed, and the obtained data were closely analyzed. Free spectra of ^1^H−^15^N MDM2 and in complex with unlabelled S100A1 were overlapped and altered or missing cross-peaks were recognized. These cross-peaks were highlighted as E25, R29, H73, F91, S92, V93, and V108 (Fig 2A). The E25, R29, H73, F91, S92, V93, and V108 residues (blue color) were also plotted onto the cartoon structure of the MDM2 protein (Fig 2B).

### Illustration of the S100A1−MDM2 complex

**Fig 3:**
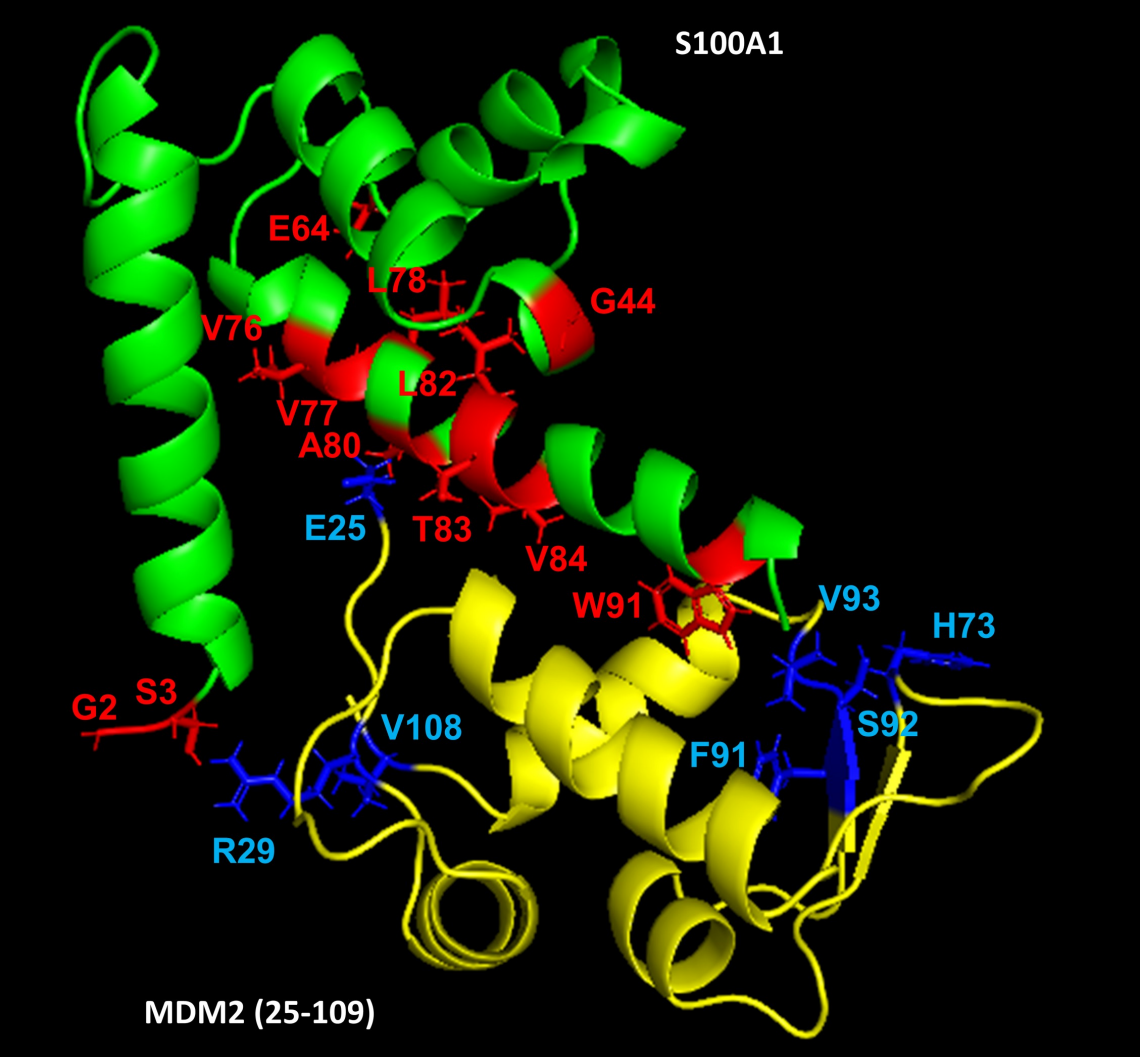
Animation displaying S100A1−MDM2 complex generated via the HADDOCK program. S100A1 is painted in green and MDM2 in yellow color. Residues neighboring the interface regions are indicated in red (from the S100A1 side) and blue (from MDM2 side), respectively.

The elemental cartoon representation of the S100A1-MDM2 complex to describe the protein-protein interaction was acquired utilizing the HADDOCK program. The input parameters for Ambiguous Interaction Constraints (AIRs) were obtained by analyzing the HSQC-NMR spectral data of S100A1 and MDM2 protein. The cross-peaks indicating changes in intensities were picked as input data for the HADDOCK program, and on completion, the resulting output created the models of S100A1-MDM2 complexes were segregated into various clusters. The reference files for the HADDOCK run were acquired from PDB ID: 2LP3 in case of calcium bound S100A1, and MDM2 input data was attained from PDB ID: 1YCR. Nearly 2000 complex models were formed in HADDOCK by rigid-body minimization, and the absolute 200 models utilizing the lowest energy results following water refinement were involved for further investigation. The generated S100A1−MDM2 complex model is portrayed in Fig 3.

In total, three conceivable binding regions were observed involving residues S3 from S100A1 with R29 from MDM2 at the first binding site; A80 and T83 from S100A1 with E25 from MDM2 at the second binding site; and W91 from S100A1 with V93 from MDM2 at the third binding site. The online PROCHECK program [68], facilitated by the European Bioinformatics Institute, was used to test the structural stereochemistry of the complex. The Ramachandran plot disclosed the occurrence of 93.2% residues in the maximum favored regions, 5.6% in additionally allowed areas, 1.2% in the allowed sector, and 0.0% in the disallowed zone (S1 Fig).

### Overlapping of S100A1-MDM2 complex with p53 (peptide)-MDM2 complex

**Fig 4:**
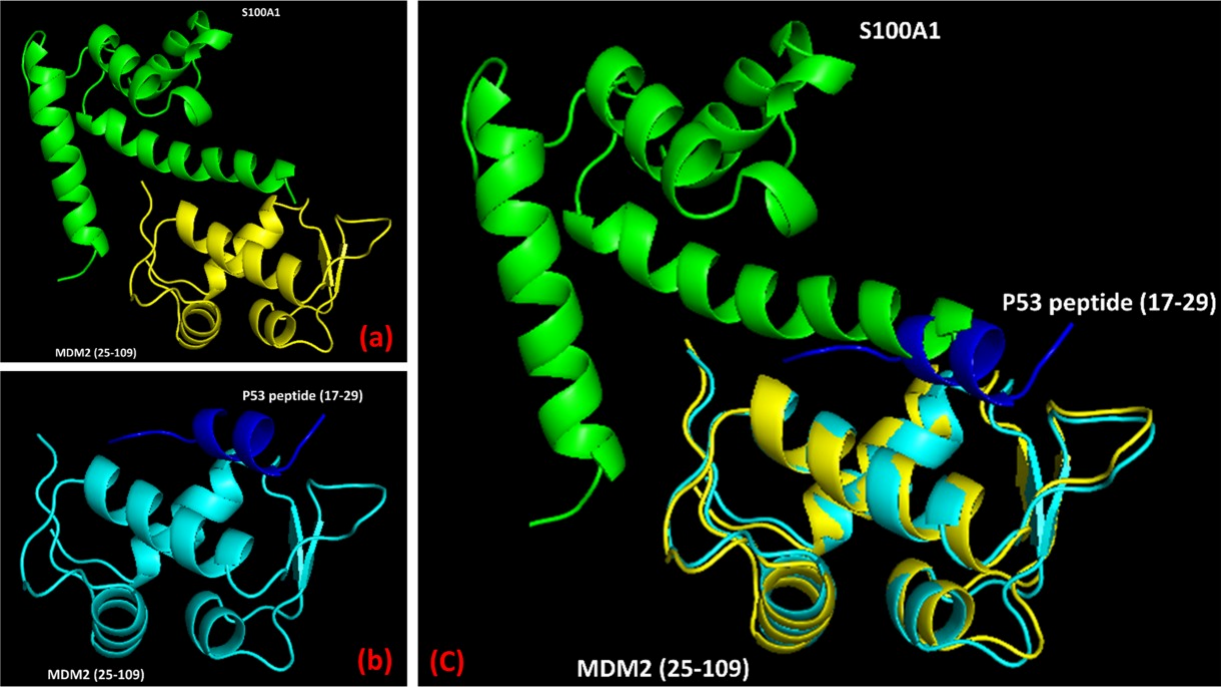
S100A1 interferes with the p53-MDM2 complex formation. (a) The complex model of the S100A1−MDM2 protein generated using the HADDOCK program. (b) The reported complex model of the p53 peptide−MDM2 domain obtained via the X-ray diffraction technique. (c) Overlay of the complex amid S100A1 (green) and the MDM2 domain (yellow), to the complex amid p53 peptide (blue) and MDM2 (yellow) showing S100A1 interference to p53-MDM2 complex.

It was noticed that MDM2 interacts through its N-terminal domain to S100A1 protein, which is depicted in the HADDOCK model (Fig 4A). Also, the X-ray diffraction technique revealed the association of MDM2 N-terminal domain to the p53 peptide (Fig 4B). Therefore, it was of high interest to overlap the MDM2 part of the S100A1-MDM2 complex with the MDM2 part of the p53 peptide-MDM2 complex to elucidate the position of S100A1 protein. The overlapping suggested that S100A1 probably interferes with the association of p53-MDM2 complex formation, thereby indicating that the S100A1 (or S100A1 derived peptide segment) could potentially block the p53-MDM2 interaction (Fig 4C).

### S100A1 could interfere with the interaction amongst p53 and MDMX

**Fig 5:**
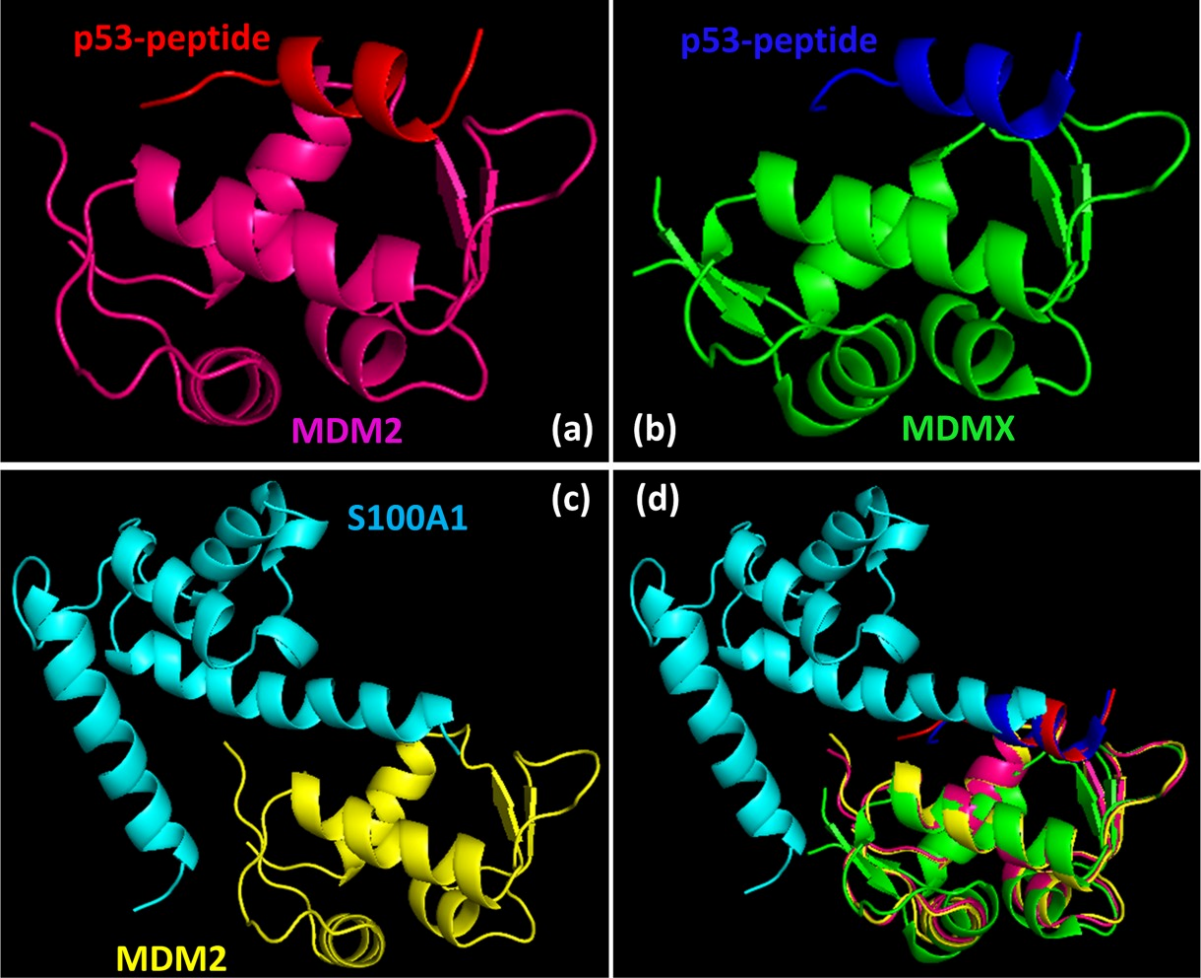
S100A1 can interfere with the interaction between p53 and MDM2/MDMX. (a) A reported complex model of p53 peptide and MDM2 N-terminal domain (PDB ID- 1YCR). (b) A reported complex model of p53 peptide and MDMX N-terminal domain (PDB ID- 3DAC). (c) A complex model of S100A1 and MDM2 N-terminal domain generated via the HADDOCK program. (d) Overlapping of the complex models indicated in a, b, and c showed possible interference of S100A1 between p53 peptide and MDM2/MDMX N-terminal domain.

We have shown here that the S100A1 interacts with MDM2 N-terminal domain and MDM2 residues important for S100A1 interaction included E25, R29, H73, F91, S92, V93, and V108. Out of these residues, F91 and V93 also participates for p53 interaction and are conserved in MDMX as well. Therefore, it is highly likely for S100A1 to interact with the MDMX N-terminal domain too as both (MDM2 and MDMX) are structurally very similar. Additionally, similar to the superimposition of S100A1-MDM2 complex with p53 (peptide)-MDM2 complex (PDB ID-1YCR), superimposition of S100A1-MDM2 complex with p53 (peptide)-MDMX complex (PDB ID-3DAC) indicated the possible intrusion of S100A1 segment to the p53 (peptide)-MDMX complex formation (Fig 5). As MDM2 and MDMX are structurally very similar and S100A1 could interfere with the interaction between p53 and MDM2/MDMX, we kept our current studies limited to the use of MDM2 N-terminal domain only.

### Interaction amongst unlabeled S100A1 and labeled p53 (1–73)

Interaction amongst p53 peptide (15–29) and MDM2 (17–125) protein indicated the presence of F19, W23, and L26 amino acid residues at the interface region, which is a part of the p53 helix (18–26). [69] Apart from p53 peptide (15–29), the p53 (1–73) region was also investigated for the binding amongst p53 (1–73) and MDM2 (17–125), thereby indicating that besides the p53 helix region (18–26), nascent turns 1 (40–45) and 2 (49–54) are furthermore able to bind to MDM2. [70]

**Fig 6:**
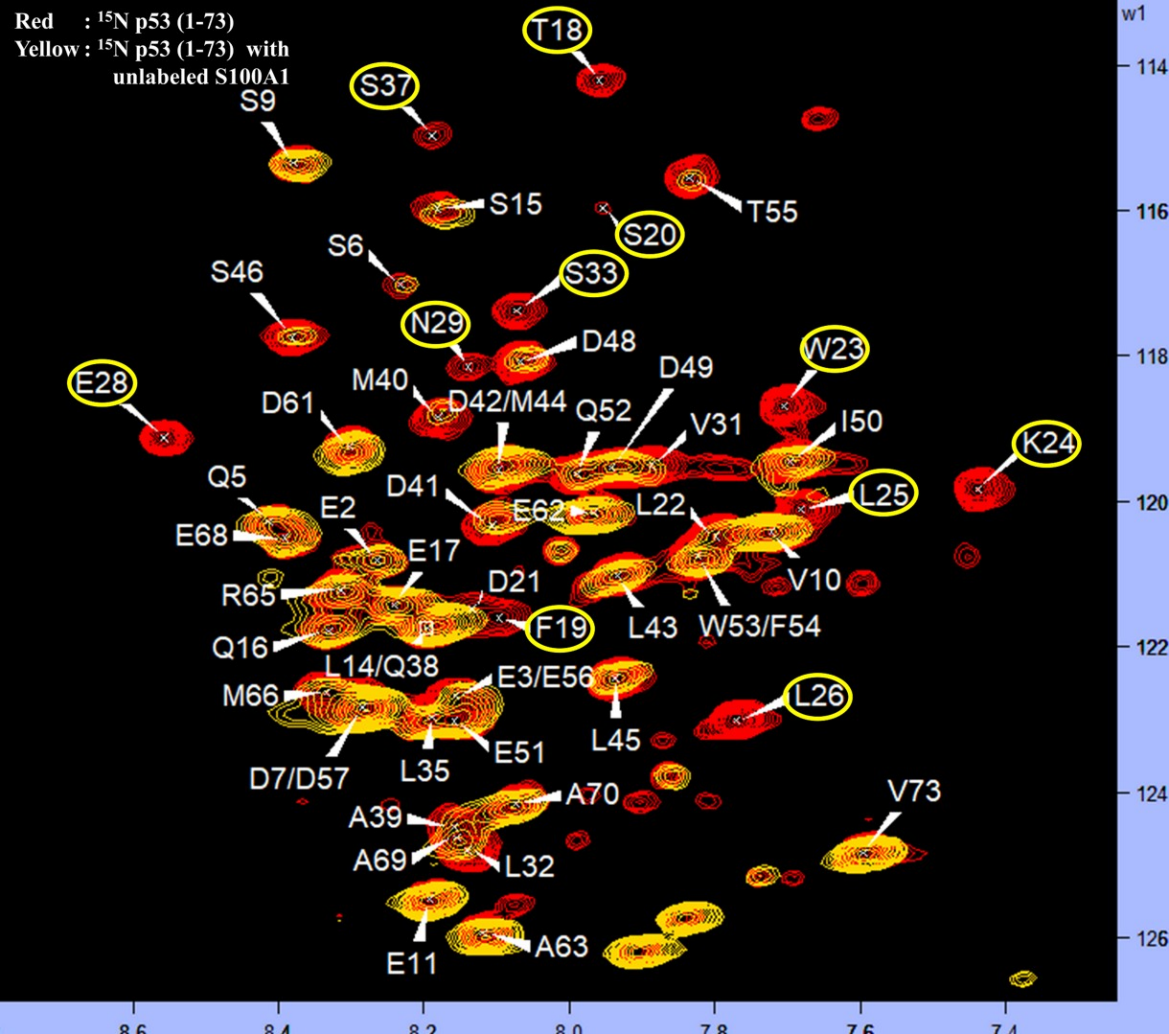
The ^1^H−^15^N HSQC spectra overlay of free ^15^N p53 (1–73) (red) and in complex with unlabelled S100A1 (yellow). Cross-peaks proving deviations in intensities are highlighted in yellow. Residues were assigned as published earlier. [70, 71].

As S100A1 interacted with the MDM2 N-terminal domain, it was of interest to analyze the p53 (1–73) residues when complexed with S100A1. Through the HSQC-NMR method, spectra obtained upon the introduction of unlabeled S100A1 to labeled p53 (1–73) (yellow color) and overlapped with labeled p53 (1–73) alone (red color), and the binding region was identified. Residues of p53 (1–73) near the interaction site, when interacted with S100A1, included T18, F19, S20, W23, K24, L25, L26, E28, N29, S33, and S37. Fig 6 demonstrates the HSQC-NMR spectra of p53 (1–73), free (red), and in complex (yellow) with S100A1. Residues exhibiting changes are highlighted.

### Interaction amongst labeled S100A1 and unlabeled p53 (1–73) and comparison of S100A1-p53 (1–73) HSQC-NMR data with S100A1-MDM2 HSQC-NMR data

**Fig 7:**
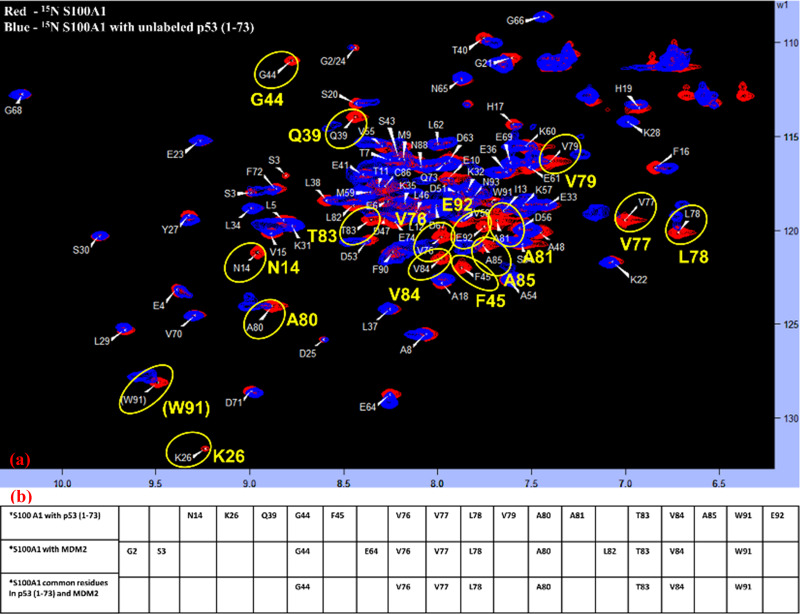
Exploration of the connected interface (S100A1/p53 (1–73)) section in S100A1. (a) The ^1^H-^15^N HSQC spectra overlay of free ^15^N S100A1 (red) and in complex with unlabelled p53 (1–73) (blue). Cross-peaks displaying variation in their magnitude are highlighted in yellow. (b) Table illustrating a list of the affected residues when ^15^N S100A1 interacts with the unlabeled p53 (1–73) and unlabeled MDM2 and also showing the common interacting residues.

To test the previous hypothesis that S100A1 could potentially interfere with the interaction amongst p53 and MDM2, we performed the interaction studies of S100A1 and p53 (1–73) domain to elucidate the S100A1 affected residues. We employed HSQC-NMR experiments to study the interacting residues. The ^15^N labeled S100A1 spectra of free form (red) was overlaid on the spectra of the ^15^N labeled S100A1 in complex through the unlabeled p53 (1–73) domain. The cross-peaks showing altered intensities were recognized and highlighted, as shown in Fig 7A.

The affected residues from S100A1 side included N14, K26, Q39, G44, F45, V76, V77, L78, V79, A80, A81, T83, V84, A85, W91, and E92. Fig 7B illustrates the affected residues in S100A1 when interacting with the p53 (1–73) and MDM2, and it also lists the common residues. Overall, the data indicated that the S100A1 residues situated at the C-terminal end were more involved during the complex formation with MDM2 and/or p53 (1–73). Therefore, it was of interest to construct the peptide derived from the S100A1 C-terminal side consisting of major residues for in-vitro studies.

### S100A1 derived peptide construction

**Fig 8:**
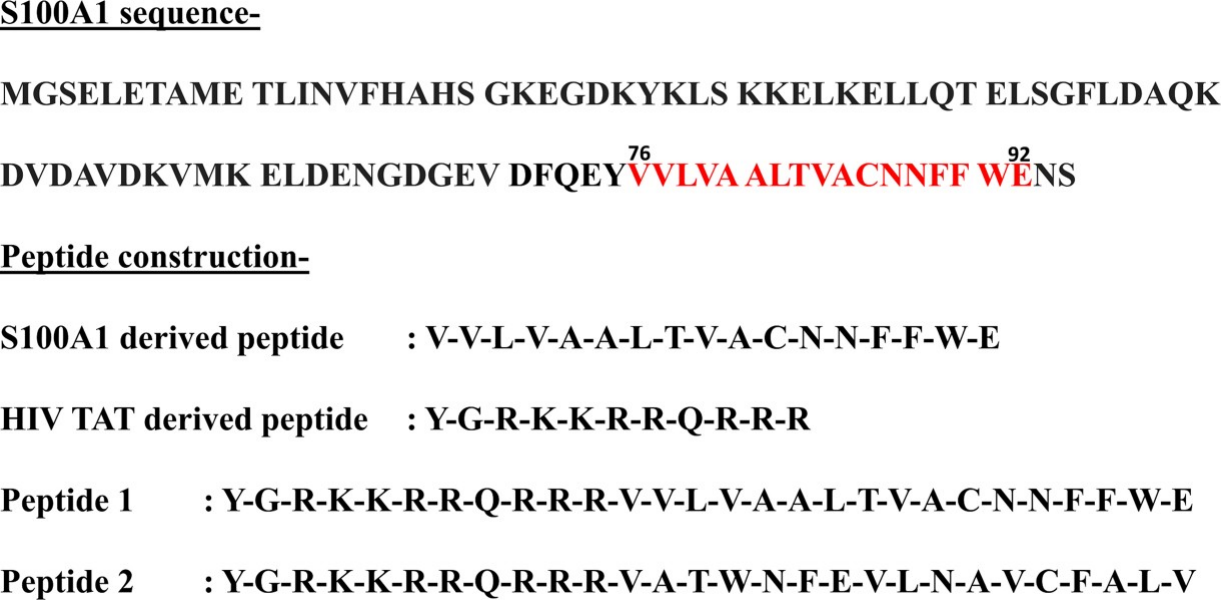
S100A1 complete sequence indicating highlighted region selected for peptide construction, attaching it to HIV-TAT peptide forming Peptide 1 and its scramble arrangement in Peptide 2.

Using the data obtained so far, it was of great interest to use the S100A1 C-terminal residues for the peptide construction. We selected residues from 76 to 92 (17 residues) for our peptide construction. We fused this 17-residue-long S100A1-derived peptide to the HIV (Human immunodeficiency virus) TAT (transcription- transactivating) segment derived from TAT protein, forming functional peptide as named Peptide 1. We also scrambled the residue sequences (76 to 92), attaching it to the HIV-TAT segment (YGRKKRRQRRR), forming reference/control peptide as named Peptide 2 with respect to Peptide 1. HIV-TAT was attached in this way because it was well reported that HIV-TAT (Sequence- YGRKKRRQRRR) is one of the popular cell-penetrating peptides used as cell transfection agents or delivery vectors, capable of transporting covalent conjugates inside the cells. [72–74] TAT segment is very well reported for its capability to deliver various cargoes into the nucleus by efficiently penetrating the cellular membrane. [75] Site-directed mutagenesis study performed by Craig A. Rosen et al. indicated that the TAT derived fusion protein containing amino acids GRKKR when fused to the N-terminal end of β-galactosidase resulted in nucleus accumulation. [76] The James Barsoum group was able to demonstrate the capability of TAT peptides to mediate cytoplasmic delivery of heterologous proteins which was independent of the cell type. [77] Interestingly, Bernard Lebleu et al. showed that the TAT fragments consisting of the whole basic region are needed for the cellular internalization and nuclear accumulation. [78] Therefore it was conceived that the TAT conjugated Peptides 1 and 2 will penetrate the cells optimally. Fig 8 illustrates the peptide construction for Peptides 1 and 2.

### CD spectra

**Fig 9:**
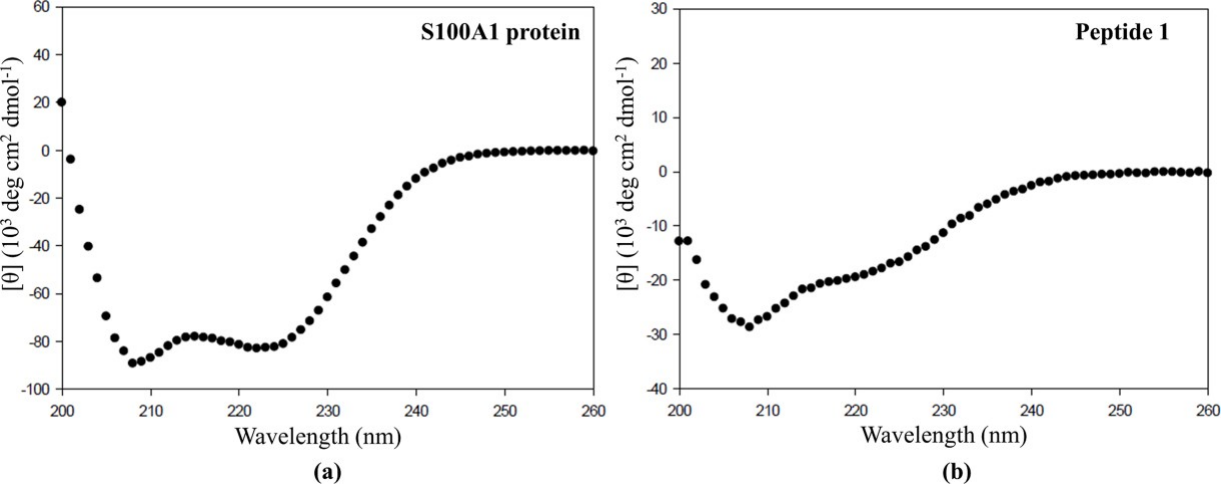
Far-UV CD spectra of (a) S100A1 protein and (b) Peptide 1.

Earlier CD studies performed by Erwann P. Loret et al. showed that the HIV-TAT alone exists as an unstructured form in the CD spectrum. [79] So, in our case, it is preferable to examine the CD spectra of Peptide 1 (contains 11 amino acids of TAT linked to the 17 amino acids of S100A1 peptide) to elucidate whether TAT has any effect on the conformation of S100A1 peptide. However, we know that the S100A1 peptide alone consists of amino-acids ranging from 76–92, which belongs to the alpha-helix region of S100A1 protein. Therefore, S100A1 peptide itself must show the α-helical character in the CD spectrum. First, we obtained the CD spectra of S100A1 protein which showed the presence of a negative dip around 208 nm and 222 nm range (Fig 9A) which is an indication of the presence of α-helix character. [80] Next, we obtained the CD spectra of Peptide 1 which showed the presence of negative dip around 208 nm and to some extend around 222 nm, as shown in Fig 9B respectively. The Peptide 1 showed the typical of α-helix character in the CD spectrum and therefore, we can say that the Peptide 1 has retained the α-helix form in the presence of TAT peptide. It means that the TAT peptide (11 amino acids) does not change the conformation of S100A1 peptide (17 amino acids).

Furthermore, Keliang Liu et al. designed and synthesized peptides consisting of endo-lysosomal membrane disrupting segment, (LLKK)3, and cell-penetrating segment (TAT sequence) to enhance gene delivery. The (LLKK)3 imparts an amphiphilic α-helical conformation to the peptide that disrupts the cellular membranes. CD spectra showed that the introduction of the TAT peptide lowered the α-helical content slightly in the conjugated peptide synthesized. [81] Altogether it can be inferred that the TAT sequence has a very minimal effect to the confirmation of the conjugated partner under consideration.

### Fluorescence experiment

We have employed the fluorescence method to study the binding affinities amongst p53-MDM2 and p53- Peptide 1. Peptide 1 and p53 contains tryptophan residues which can be selectively excited by using the excitation wavelength of 295 nm or above. [82] In our fluorescence experiments, we could not notice the emission spectra of the tryptophan residue of Peptide 1 by selective excitation at 295 nm. However, tryptophan residues belonging to the p53 does show emission spectra by selective excitation at 295 nm. We noticed that the p53 emission spectra increased upon binding with its respective partners (MDM2 and Peptide 1). This could be explained that the binding of p53 with its respective partners makes tryptophan residues more exposed to the surface which is otherwise partially exposed and/or less accessible.

**Fig 10:**
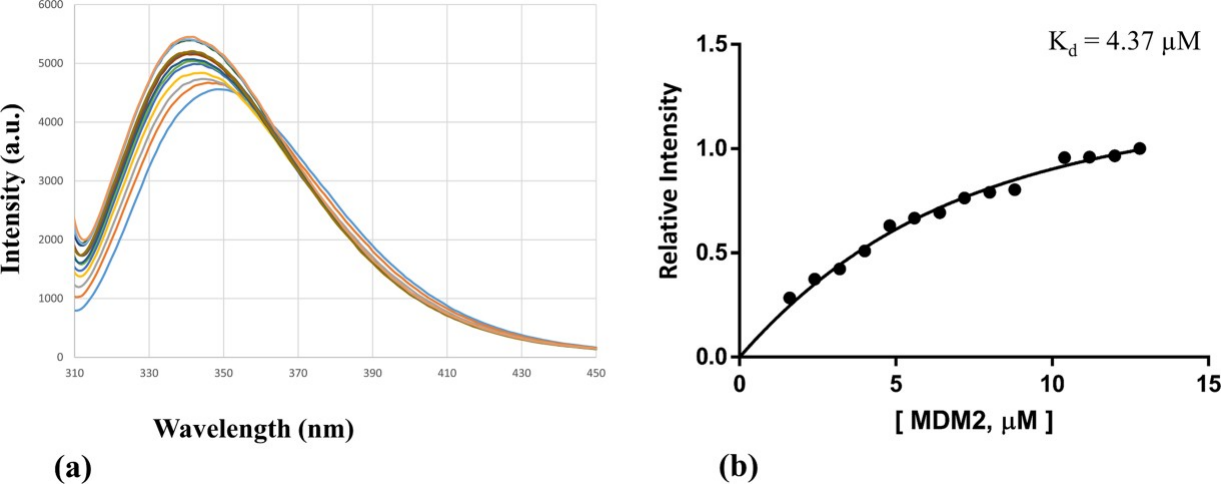
The p53-MDM2 interaction was monitored via fluorescence spectroscopy. (a) Changes in the fluorescence emission spectra of p53 upon the addition of increasing concentration of MDM2 in the micro-molar range was observed. (b) Relative intensity versus the MDM2 total concentration.

We first studied the interaction between p53 (1–73) and MDM2 (17–125), and the non-linear curve fitting of the relative intensity versus the MDM2 total concentration resulted in a dissociation constant, K_d_, of around 4.37 μM as shown in the following Fig 10.

**Fig 11:**
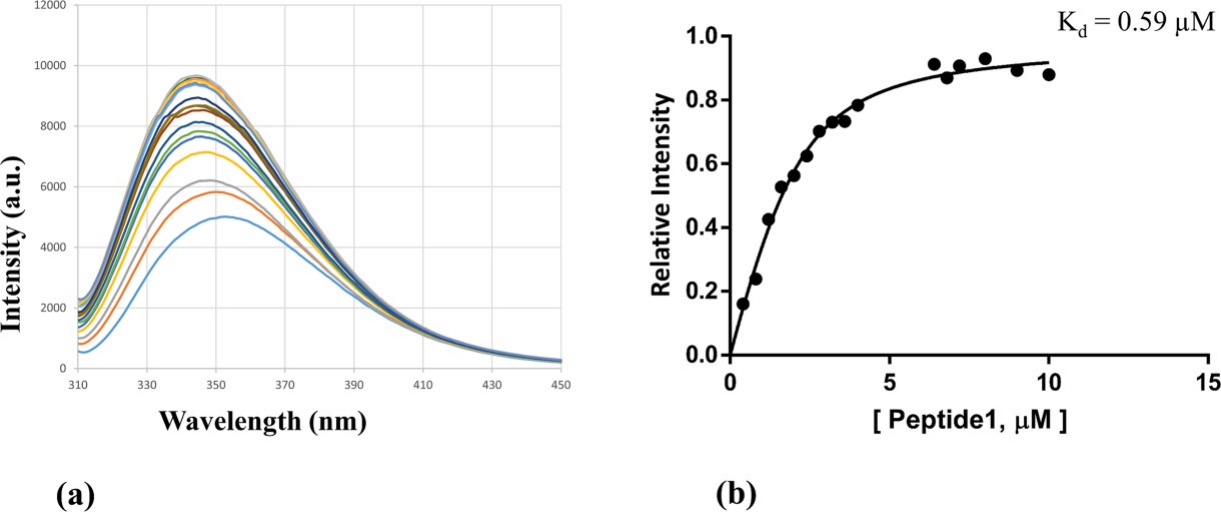
The p53-Peptide 1 interaction was monitored via fluorescence spectroscopy. (a) Changes in the fluorescence emission spectra of p53 upon the addition of increasing concentration of Peptide 1 in the micro-molar range was observed. (b) Relative intensity versus the Peptide 1 total concentration.

Next, we studied the interaction between p53 (1–73) and Peptide 1, and the non-linear curve fitting of the relative intensity versus the Peptide 1 total concentration resulted in a dissociation constant, K_d_, of around 0.59 μM as shown in the following Fig 11.

From the above fluorescence data, it can be observed that the binding between p53 and Peptide 1 is stronger (K_d_ = 0.59 μM) than the binding between p53 and MDM2 (K_d_ = 4.37 μM). So there is a possibility that the Peptide 1 could interfere with the p53-MDM2 association.

### Competitive binding experiment by NMR

**Fig 12:**
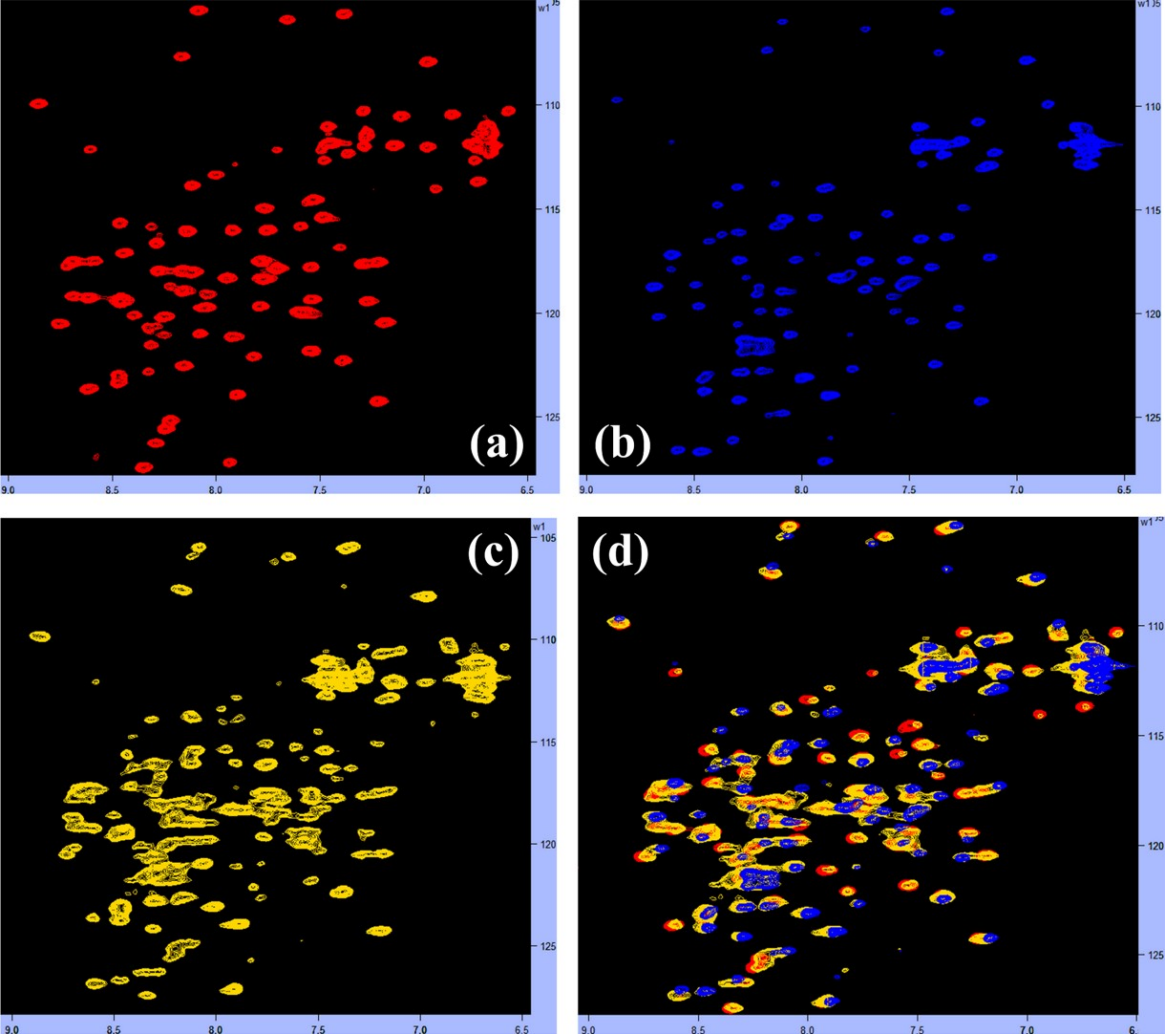
Competitive binding experiment. (a) The ^1^H-^15^N HSQC spectra of ^15^N MDM2 in complex with unlabeled Peptide 1 (red) in a 1:1 ratio. (b) The ^1^H-^15^N HSQC spectra of ^15^N MDM2 in complex with unlabeled p53 (blue) in a 1:1 ratio. To the same mixture, we added the Peptide 1 in a 1:1 ratio and obtained the spectrum as shown in (c). Then the spectra obtained in (b) and (c) is overlapped to the spectra indicated in (a) and the resulting overlapped spectra are shown in (d).

We carried out the HSQC-NMR competitive binding experiment to see if Peptide 1 could interfere with the MDM2-p53 interaction. We first added the unlabeled Peptide 1 to the ^15^N labelled MDM2 in a 1:1 ratio and obtained the HSQC spectrum as shown in Fig 12A. Next, we added the unlabeled p53 to the ^15^N labelled MDM2 in a 1:1 ratio and obtained the HSQC spectrum as shown in Fig 12B. Then to the same mixture (^15^N labelled MDM2 complexed with unlabeled p53) we added the unlabeled Peptide 1 in a 1:1 ratio and obtained the HSQC spectrum as shown in ig 12c. Afterward, we overlapped these 3 HSQC spectra together and showed in Fig 12D. The spectral analysis indicated that several ^15^N labeled MDM2 peaks were shifted and/or disappeared after the interaction with p53 (Fig 12B). Upon the addition of Peptide 1, most of the shifted and/or disappeared ^15^N labeled MDM2 peaks reappear (Fig 12C) and their position matches/coincide with the ^15^N labeled MDM2 peaks when it interacted with the Peptide 1 (Fig 12A). The above HSQC-NMR competition experiments indicate that indeed Peptide 1 replaces p53 from the MDM2-p53 complex. It means that Peptide 1 could interfere successfully with the MDM2-p53 association.

### WST-1 assay

A WST-1 centered cell propagation experiment was used to study the effect of S100A1-derived Peptide 1 and its scramble version Peptide 2 to the cancer cell line. The widely known cell line MCF-7 (HTB-22, Breast carcinoma) was used in our functional assay as they express wild-type p53 as well as MDM2 and MDMX. [83] Cells were raised in the presence or absence of Peptide 1 and/or Peptide 2 consisting of varying concentrations of 5, 10, and 20 μM. After a 48-hour incubation period, a specified volume of WST-1 reagent was added at 4 hours before harvest, and the absorbance was determined at 450 nm. Viable cells were normalized by the absorbance from the control cells, which is only the cells without any additives. Both the TAT-fused peptides, 1 and 2, were able to penetrate the cell membrane and reached the area of cytosol and nucleus compartments.

The WST-1 experiment was done after 48 hours of incubation of the cells with the Peptides 1 and 2 as on average, the MCF-7 cells population doubling time is about 24 hours. [84] Robert L. Sutherland et al. studied the cell proliferation kinetics of the MCF-7 cells in the monolayer culture and found that after crossing the lag phase, the cell population multiplies every 24 hours on average. Therefore, to notice a considerable change in the cell population, we carried out the WST-1 assay after 48 hours of incubation, which is reasonable.

**Fig 13:**
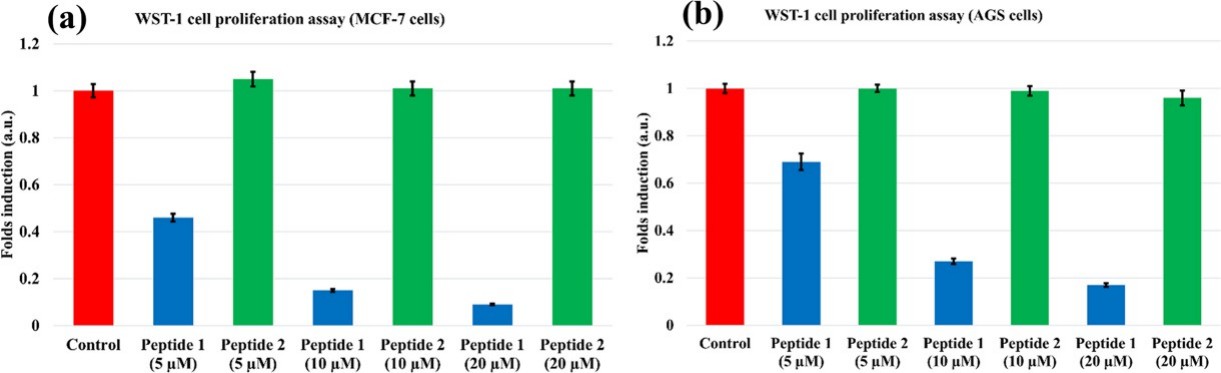
Analysis of WST-1 assay. The (a) MCF-7 and (b) AGS cells were treated with 5, 10, and 20 μM concentrations of Peptide 1 (blue) and Peptide 2 (green). The comparative cell counts after successive treatment with Peptides 1 and 2 are shown as fold inductions with the control (red) as serum-free media alone.

Cells treated with Peptide 1 showed a significant decrease in viable cell counts when compared to the control, and the Peptide 2 treated cells were comparable to the control cells. Additionally, we have chosen another gastric cancer cell line, AGS, with wild type p53 and high MDM2 expression for proving our concept. Similar results were observed that treatment with Peptide 1 significantly reduced the cell numbers of AGS cells after 48h. However, treatment with Peptide 2 did not have the obvious effect. Fig 13 displays the effect of Peptide 1 and Peptide 2 on the (a) MCF-7 cells and (b) AGS cells.

The work done by Sonia Aroui et al. performing cytotoxic studies pertaining to TAT peptide alone on various cell lines including MCF-7 indicated that up to 5 μM concentration, TAT peptide is non-toxic to the cell lines and above 5 μM concentration, it showed limited cytotoxicity in the range of 11–22%. [85] Even in our case, the TAT segment is unaltered and only the amino-acid sequences from the S100A1 C-terminal part (residues, 76–92) have been scrambled in Peptide 2 and kept original in Peptide 1. In the WST-1 assay, Peptide 2 has shown no considerable effect on the cells with concentration even up to 20 μM. So, overall as observed earlier, in our case too we predict that the TAT sequence will impart minimal or slight effect on the cells and the conjugated TAT has shown no effect on the cells.

Moreover, the Roland Brock group detected the cytoplasmic fluorescence in the HeLa cells for the N-terminal fluorescein tagged TAT peptide even after five hours indicating that TAT peptide alone is quite stable. [86] Furthermore, Sonia Aroui et al. showed the presence of Dox-TAT conjugate, after two hours, inside the cells by the fluorescence tag by utilizing five cell lines (CHO, HUVEC, MDA-MB 231, MCF7 and NG108 cells). [85] The TAT conjugates were easily detected via fluorescence inside the cells even after the four hours’ treatment and the twelve hours’ treatment as shown previously. [81, 87] So, definitely Peptides 1 and 2 are quite stable and therefore has shown the results in the WST-1 assay after 48 hours of incubation. Though we cannot say exactly for how long Peptides 1 and 2 are stable inside the cell as for now, it can be deduced from the WST-1 assay results that the reduction in the viable cell-count is imparted primarily by the Peptide 1 treatment, compared to Peptide 2, which is an indication of its reasonable stability.

### Western blotting

**Fig 14:**
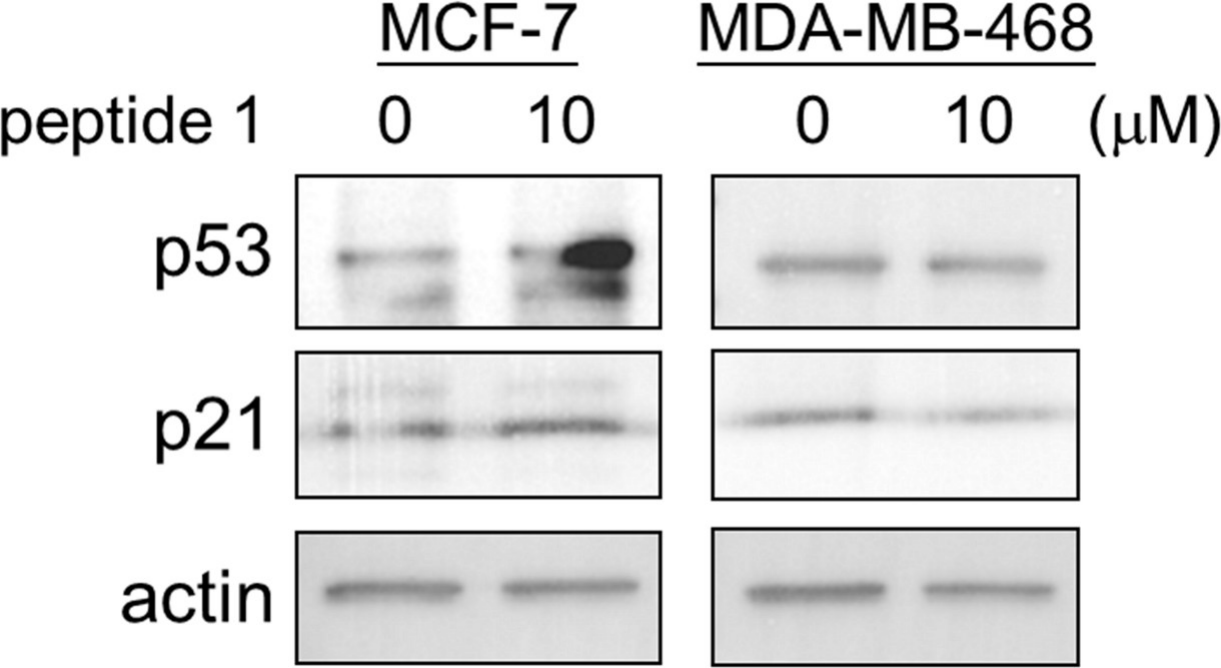
The MCF-7 and/or MDA-MB-468 cells were treated with or without 10 μM Peptide 1 for 48h. After the treatment, cells were collected and lysed. The protein levels of p53, its down-stream p21, and actin were examined by Western blotting. The Actin was used as an internal control.

To elucidate the Peptide 1 specificity for the p53 pathway and its predictable role to interfere with the MDM2-p53 interaction, we carried out the Western blotting experiment. In addition, we alternatively used two breast cancer cell lines, MCF-7 (expressing wild type p53) and MDA-MB-468 (expressing mutant p53) for comparison. The protein levels of p53 and its down-stream p21 were determined by Western blotting. The results showed that both, p53 and p21, were induced after the Peptide 1 treatment for 48h in MCF-7 cells, but not in MDA-MB-468 cells, indicating that Peptide 1 could activate the p53-p21 pathway in breast cancer cells with wild type p53. Also, it can be inferred that the Peptide 1 could successfully interfere with the MDM2-p53 interaction thereby stabilizing the p53 as its concentration increased in MCF-7 cells after 10 μM Peptide 1 treatment for 48h. Fig 14 displays the increased protein level of p53 and p21 after the Peptide 1 treatment for 48h in MCF-7 cells, but not in MDA-MB-468 cells.

### Cell cycle analysis

**Fig 15:**
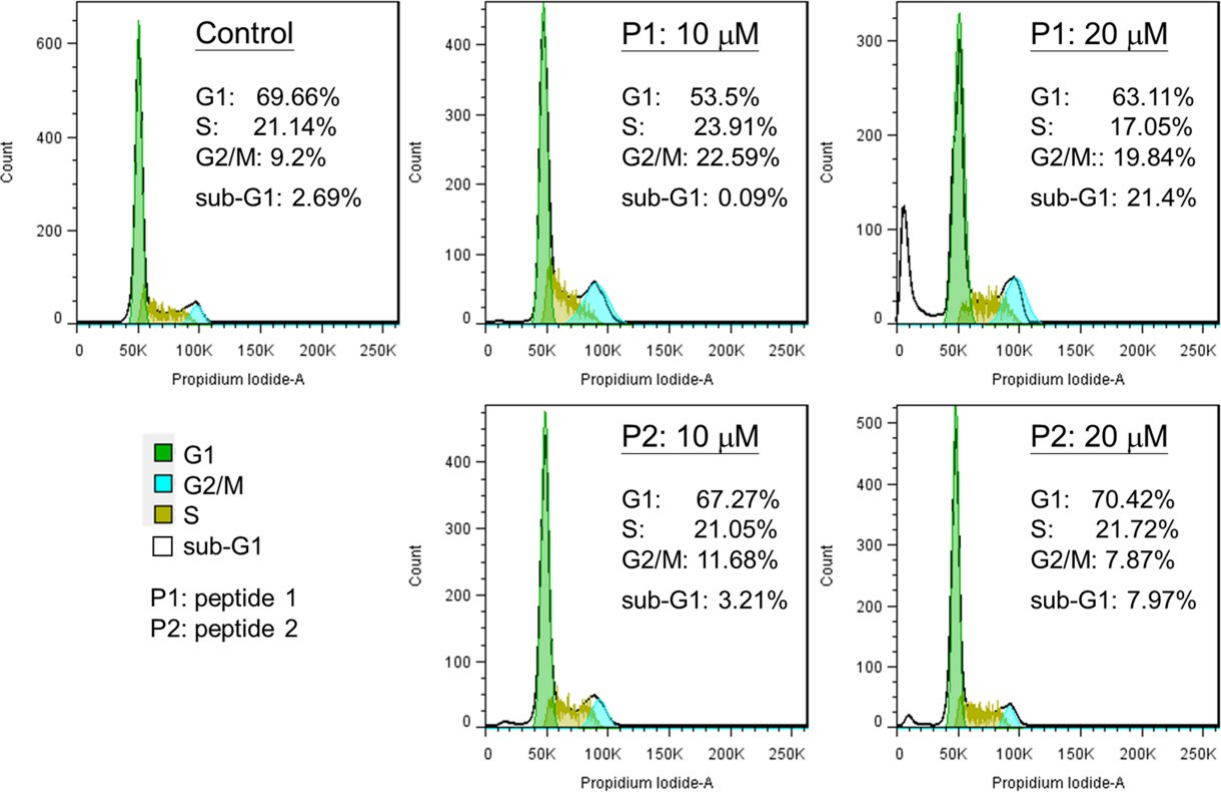
MCF-7 cells were treated with 0, 10, or 20 μM of Peptide 1 or Peptide 2 for 48h. Subsequently, cells were collected, fixed with 70% ethanol, and stained with 10 μg/ml propidium iodide. The cell cycle distribution was analyzed by a flow cytometer.

In order to elucidate the potential role of Peptide 1 to activate p53 function in cancer cells, we carried out cell cycle analysis. We have determined the effects of peptide 1 and 2 on cell cycle distribution and apoptotic cell death (sub-G1 population) in MCF-7 cells by a flow cytometry analysis. As shown in Fig 15, treatment of peptide 1 resulted in cell cycle arrest at G2/M phase, and also induced apoptotic cell death at higher concentration (20 μM). However, treatment of peptide 2 did not have significant alternations in cell cycle contribution and apoptotic cell death. Taken together, the results suggest that disruption of the interaction of p53 and MDM2 by Peptide 1 could activate normal p53 functions, leading to cell cycle arrest and apoptotic cell death in cancer cells.

## Conclusion

**Fig 16:**
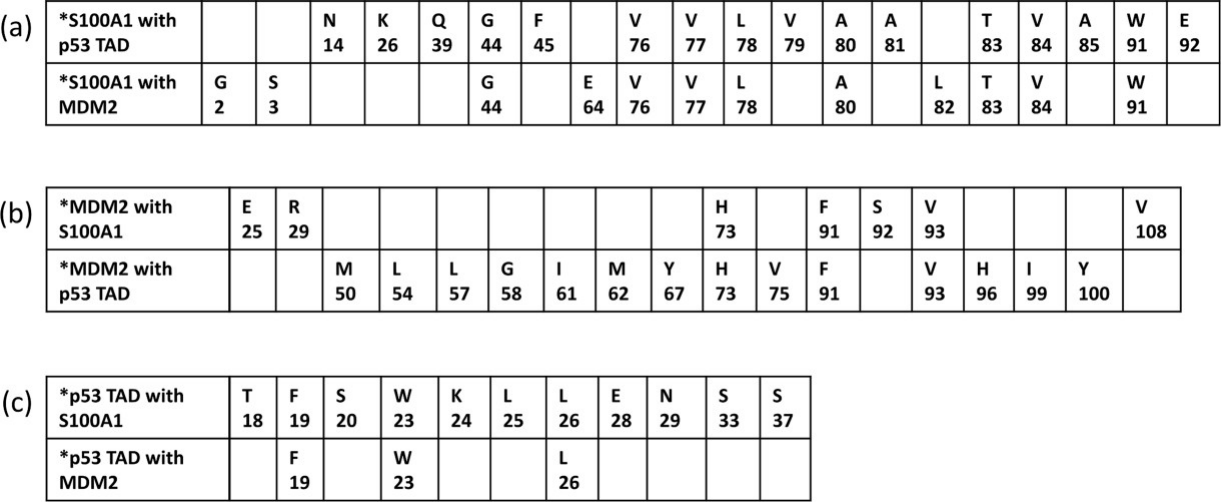
Table summarizing the list of interacting residues when (a) S100A1 interacts with p53 TAD and MDM2, (b) MDM2 interacts with S100A1 and p53 TAD, and (c) p53 TAD interacts with S100A1 and MDM2.

The S100 group of proteins that interact with p53 and MDM2 were previously studied by employing different techniques. [49–52] It has been well established that MDM2 via its N-terminal domain binds to the N-terminal domain of p53 and negatively regulates its activity. Blocking between p53 and MDM2 interaction via various means, such as synthetic peptides or customized drug molecules, could potentially reactivate the function of p53 in cancerous cells containing wild type variant of p53. [39, 88] In this work, we studied the interactions amongst S100A1 and MDM2, and between S100A1 and p53-TAD by employing HSQC-NMR spectroscopy to identify the residues at the interface regions of these proteins. Interacting residues of p53 (peptide) and MDM2 were already revealed previously. [69] List of the residues near the interface regions in S100A1 when interacted with MDM2 and/or p53, MDM2 when interacted with S100A1 and/or p53, and p53 when interacted with S100A1 and/or MDM2 are summarized in Fig 16 as tabular form and mentioned as a, b, and c for reference.

It can be observed that the S100A1 consists of a common segment comprising similar residues when interacted with MDM2 or p53 indicating that the S100A1 may bind possibly to either MDM2 or p53 at any given time. The same can be noticed for MDM2 and p53. This may be the possible reason for not forming the ternary complex amongst S100A1, MDM2, and p53 as mentioned earlier.

We used docking protocols and generated a complex of S100A1 and MDM2. We overlapped this complex to the previously reported complex of MDM2 with p53 peptide, which was derived from the p53 (1–73) region. We observed the probability of blocking MDM2-p53 interaction via S100A1 protein from its C- terminal end. This C- terminal end region in S100A1 protein would constitute similar residues that are affected when interacting with MDM2 or p53 (1–73). We hypothesize the possibility of a peptide derived from the S100A1 C-terminal end region, which could interfere with the MDM2-p53 interaction. We synthesized this peptide fused with TAT peptide. For simplicity, this fusion peptide was named Peptide 1, and its scramble version was named Peptide 2. Then, we performed the fluorescence assay and the competitive binding experiment. The data obtained from the fluorescence experiment and the competitive binding experiment showed that Peptide 1 could successfully interfere with the MDM2-p53 interaction. Afterward, we carried out a cell proliferation assay using MCF-7 and AGS cancer cells and tested the potential of Peptide 1 in-vitro. The results indicated that the Peptide 1 treated viable cells population was reduced significantly when compared to the control cells and Peptide 2 treated cells. This could be possible due to the Peptide 1 interference to the MDM2-p53 interaction. Further, Western blotting and cell cycle profiling indicated the credible role of Peptide 1 towards the p53 pathway activation thereby stabilizing p53 and disrupting the MDM2-p53 interaction. Overall, based on our results obtained so far, we could also say the possibility of MDM2 being blocked by S100A1 at a 1:1 ratio is highly possible. Also, S100A1 is highly likely to interact with the MDMX N-terminal domain as MDM2 and MDMX are structurally very similar. Therefore, S100A1 could interfere with the MDMX-p53 interaction at the same or similar stoichiometry as well. Thus, we hypothesize that S100A1 could disrupt the MDM2-p53 interaction, restricts the MDM2 function, reactivate the p53 function and reduces cellular proliferation. However, more experimental work needs to be done further to elucidate the specific role of S100A1 and its mechanism of action. The data presented here are very encouraging and elucidate the possibility of more customized development of S100A1 as a drug molecule against cancer development.

## Supporting information

S1 Fig The Ramachandran plot. The online PROCHECK program was used to test the structural stereochemistry of the complex shown in Fig 3. The Ramachandran plot disclosed the occurrence of 93.2% residues in the maximum favoured regions, 5.6% in additionally allowed areas, 1.2% in allowed sector, and 0.0% in the disallowed zone.
(DOCX)

S2 Fig The MDM2 protein purity and the mass confirmation. (a) SDS-PAGE displaying the purified MDM2 protein corresponding to the molecular weight of 12.9 kDa. S represents the crude MDM2-GST tag fusion protein, F1 is the flow and E1 is the elute collected before enzyme digestion, AED represents the E1 sample incubated with the PreScission protease enzyme for the 16 hours’ enzyme digestion, F2 represents the cleaved MDM2 protein (12.9 kDa) collected in flow after enzyme digestion, E2 represents the mixture of any remained fusion protein (39.3 kDa) together with cleaved GST tag (26.4 kDa), and M indicates the marker. (b) Confirmation of the molecular weight of the cleaved MDM2 protein via ESI-MS analysis.
(DOCX)

S3 Fig The S100A1 protein purity and the mass confirmation. (a) SDS-PAGE showing purified S100A1 protein near the molecular weight of 10.5 kDa. S represents the crude S100A1 protein, F1 and E1 represents the flow and elute collected through the Q-Sepharose column, F2 represents the flow collected upon the introduction of E1 into the Phenyl-Sepharose column and E2 indicates the elute collected over the Phenyl-Sepharose column representing the S100A1 protein (10.5 kDa). (b) Confirmation of the molecular weight of the purified S100A1 protein via ESI-MS analysis.
(DOCX)

S4 Fig The p53 (1–73) protein purity and the mass confirmation. (a) SDS-PAGE demonstrating the various fractions of p53 (1–73) protein collected through the NiNATA Superflow resin column. S represents the crude sample loaded onto the column; LB, WB, and EB indicate the elute fractions collected by employing lysis buffer, wash buffer, and the elution buffer. EB fraction contained the p53-Histidine tag fusion protein (10.5 kDa) which comes above the 15 kDa band before enzyme digestion. Following enzyme digestion with Thrombin, fusion protein after enzyme digestion was loaded onto the Superdex 75 (SEC) column. (b) SDS-PAGE indicating the fractions (1 to 7) collected after enzyme digestion and cleaved p53 (1–73) protein (8.6 kDa) is observed (fraction, 5 and 6) close to the 15 kDa band, though fraction 7 has less protein concentration. (c) Confirmation of the molecular weight of the cleaved p53 (1–73) protein via ESI-MS analysis.
(DOCX)
