# Supplementary material for: S100A1 blocks the interaction between p53 and mdm2 and decreases cell proliferation activity
Source: PLoS One. 2020 Jun 4;15(6):e0234152. doi: 10.1371/journal.pone.0234152 (PMC7272100; doi:10.1371/journal.pone.0234152)
Supplement: S1 Fig — The online PROCHECK program was used to test the structural stereochemistry of the complex shown in Fig 3. The Ramachandran plot disclosed the occurrence of 93.2% residues in the maximum favoured regions, 5.6% in additionally allowed areas, 1.2% in allowed sector, and 0.0% in the disallowed zone. (DOCX) [file pone.0234152.s001.docx]

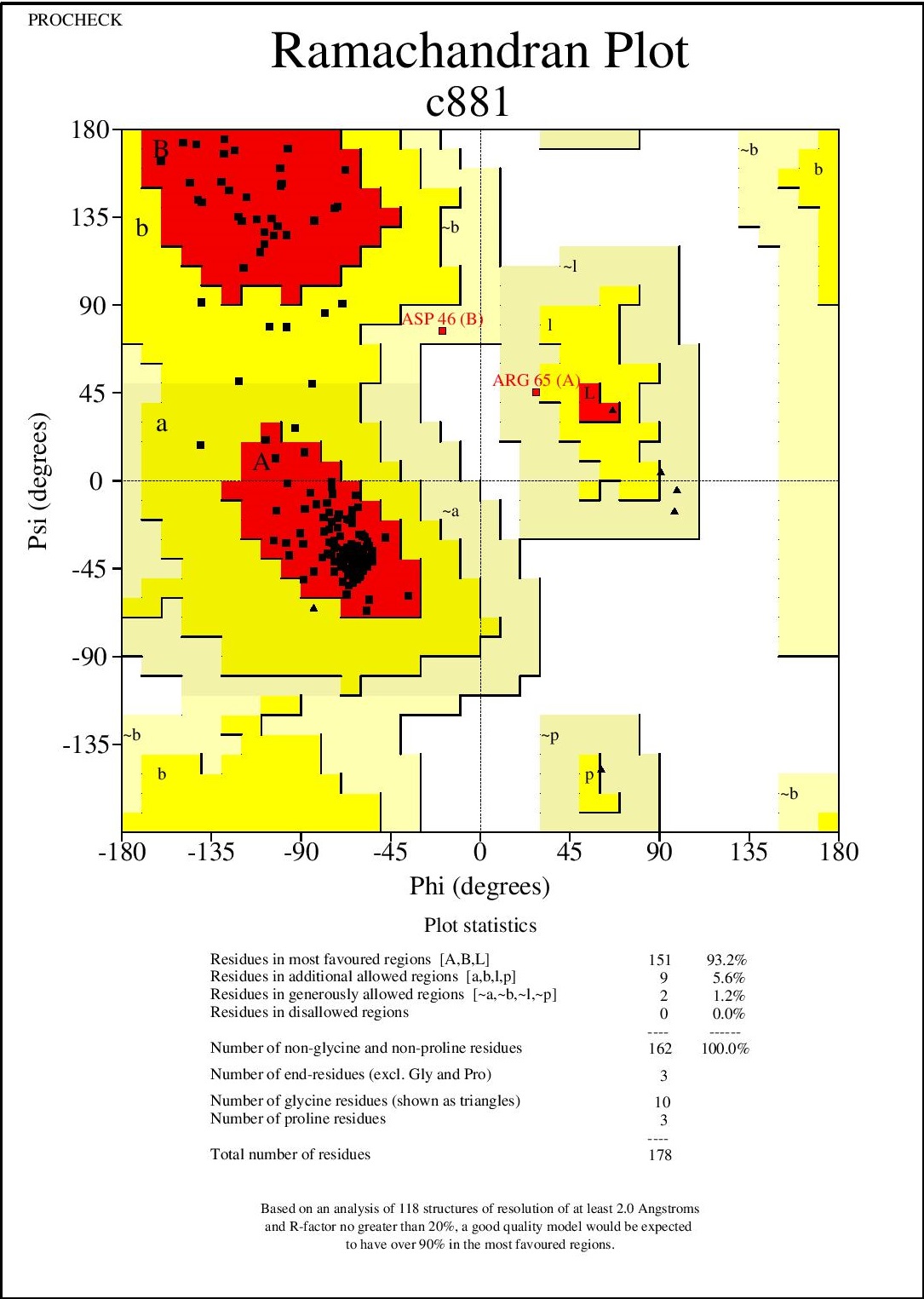


**S1 Fig. The Ramachandran Plot.** The online PROCHECK program was used to test the structural stereochemistry of the complex shown in figure 3. The Ramachandran plot disclosed the occurrence of 93.2% residues in the maximum favored regions, 5.6% in additionally allowed areas, 1.2% in allowed sector, and 0.0% in the disallowed zone.
