# Supplementary material for: S100A1 blocks the interaction between p53 and mdm2 and decreases cell proliferation activity
Source: PLoS One. 2020 Jun 4;15(6):e0234152. doi: 10.1371/journal.pone.0234152 (PMC7272100; doi:10.1371/journal.pone.0234152)
Supplement: S2 Fig — (a) SDS-PAGE displaying the purified MDM2 protein corresponding to the molecular weight of 12.9 kDa. S represents the crude MDM2-GST tag fusion protein, F1 is the flow and E1 is the elute collected before enzyme digestion, AED represents the E1 sample incubated with the PreScission protease enzyme for the 16 hours’ enzyme digestion, F2 represents the cleaved MDM2 protein (12.9 kDa) collected in flow after enzyme digestion, E2 represents the mixture of any remained fusion protein (39.3 kDa) together with cleaved GST tag (26.4 kDa), and M indicates the marker. (b) Confirmation of the molecular weight of the cleaved MDM2 protein via ESI-MS analysis. (DOCX) [file pone.0234152.s002.docx]

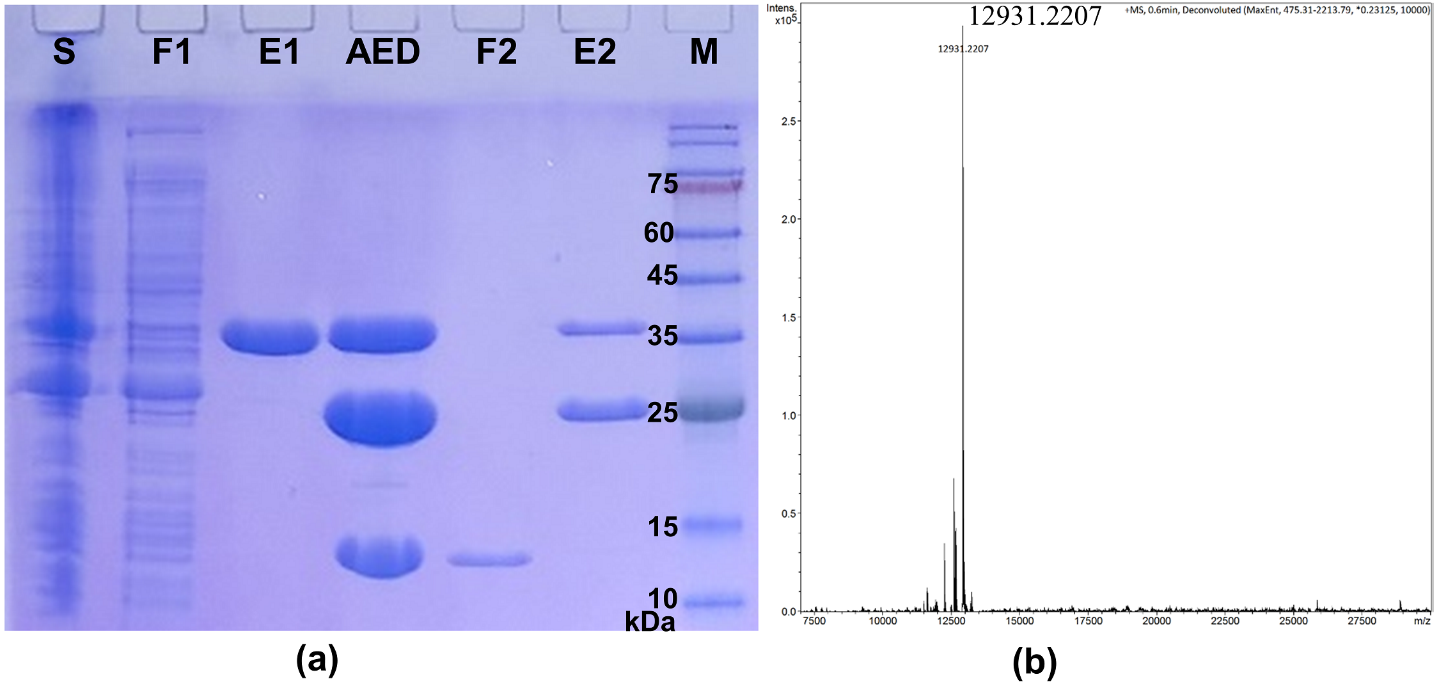


**S2 Fig.** **The MDM2 protein purity and the mass confirmation.** (a) SDS-PAGE displaying the purified MDM2 protein corresponding to the molecular weight of 12.9 kDa. S represents the crude MDM2-GST tag fusion protein, F1 is the flow and E1 is the elute collected before enzyme digestion, AED represents the E1 sample incubated with the PreScission protease enzyme for the 16 hours’ enzyme digestion, F2 represents the cleaved MDM2 protein (12.9 kDa) collected in flow after enzyme digestion, E2 represents the mixture of any remained fusion protein (39.3 kDa) together with cleaved GST tag (26.4 kDa), and M indicates the marker. (b) Confirmation of the molecular weight of the cleaved MDM2 protein via ESI-MS analysis.
