# Supplementary material for: S100A1 blocks the interaction between p53 and mdm2 and decreases cell proliferation activity
Source: PLoS One. 2020 Jun 4;15(6):e0234152. doi: 10.1371/journal.pone.0234152 (PMC7272100; doi:10.1371/journal.pone.0234152)
Supplement: S3 Fig — (a) SDS-PAGE showing purified S100A1 protein near the molecular weight of 10.5 kDa. S represents the crude S100A1 protein, F1 and E1 represents the flow and elute collected through the Q-Sepharose column, F2 represents the flow collected upon the introduction of E1 into the Phenyl-Sepharose column and E2 indicates the elute collected over the Phenyl-Sepharose column representing the S100A1 protein (10.5 kDa). (b) Confirmation of the molecular weight of the purified S100A1 protein via ESI-MS analysis. (DOCX) [file pone.0234152.s003.docx]

**
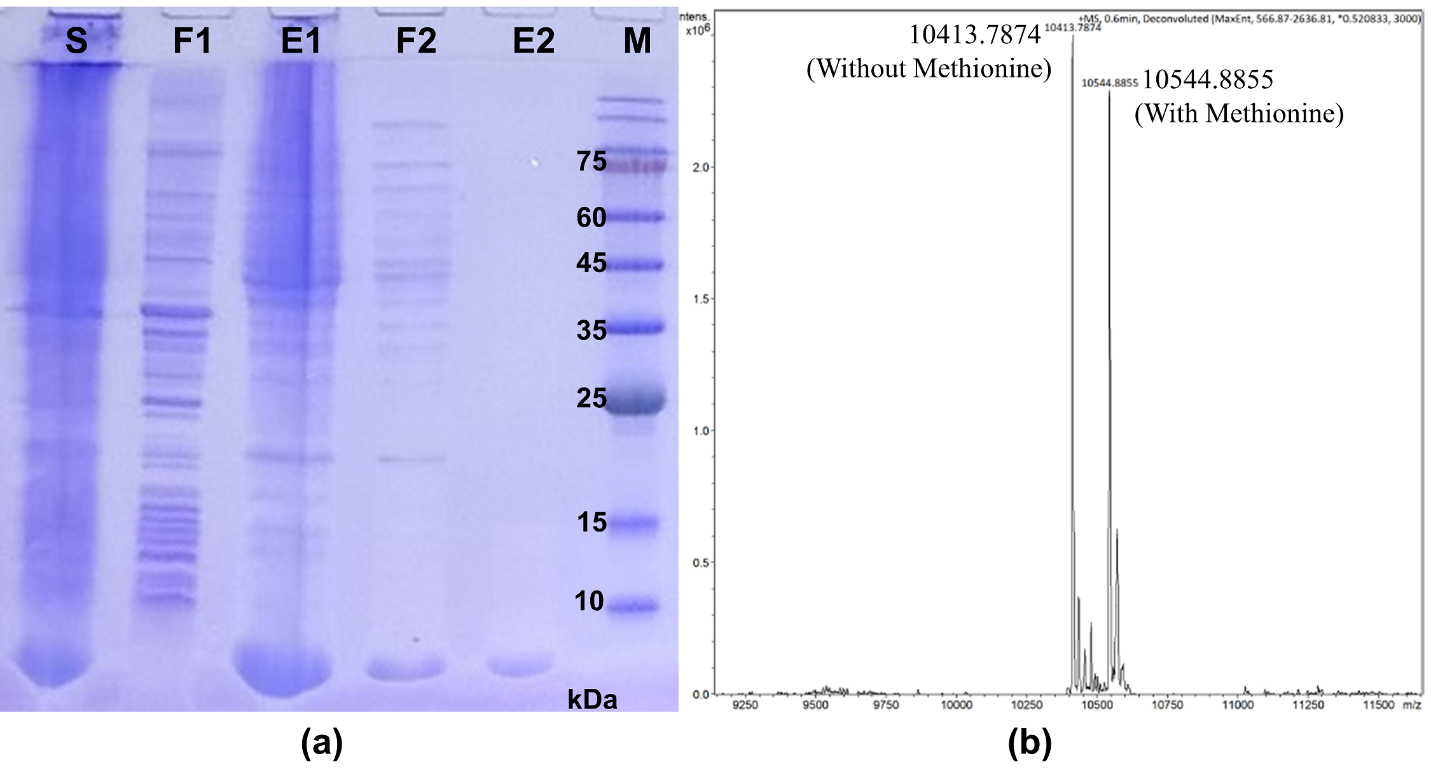
**

**S3 Fig.** **The S100A1 protein purity and the mass confirmation.** (a) SDS-PAGE showing purified S100A1 protein near the molecular weight of 10.5 kDa. S represents the crude S100A1 protein, F1 and E1 represents the flow and elute collected through the Q-Sepharose column, F2 represents the flow collected upon the introduction of E1 into the Phenyl-Sepharose column and E2 indicates the elute collected over the Phenyl-Sepharose column representing the S100A1 protein (10.5 kDa). (b) Confirmation of the molecular weight of the purified S100A1 protein via ESI-MS analysis.
