# Supplementary material for: S100A1 blocks the interaction between p53 and mdm2 and decreases cell proliferation activity
Source: PLoS One. 2020 Jun 4;15(6):e0234152. doi: 10.1371/journal.pone.0234152 (PMC7272100; doi:10.1371/journal.pone.0234152)
Supplement: S4 Fig — (a) SDS-PAGE demonstrating the various fractions of p53 (1–73) protein collected through the NiNATA Superflow resin column. S represents the crude sample loaded onto the column; LB, WB, and EB indicate the elute fractions collected by employing lysis buffer, wash buffer, and the elution buffer. EB fraction contained the p53-Histidine tag fusion protein (10.5 kDa) which comes above the 15 kDa band before enzyme digestion. Following enzyme digestion with Thrombin, fusion protein after enzyme digestion was loaded onto the Superdex 75 (SEC) column. (b) SDS-PAGE indicating the fractions (1 to 7) collected after enzyme digestion and cleaved p53 (1–73) protein (8.6 kDa) is observed (fraction, 5 and 6) close to the 15 kDa band, though fraction 7 has less protein concentration. (c) Confirmation of the molecular weight of the cleaved p53 (1–73) protein via ESI-MS analysis. (DOCX) [file pone.0234152.s004.docx]

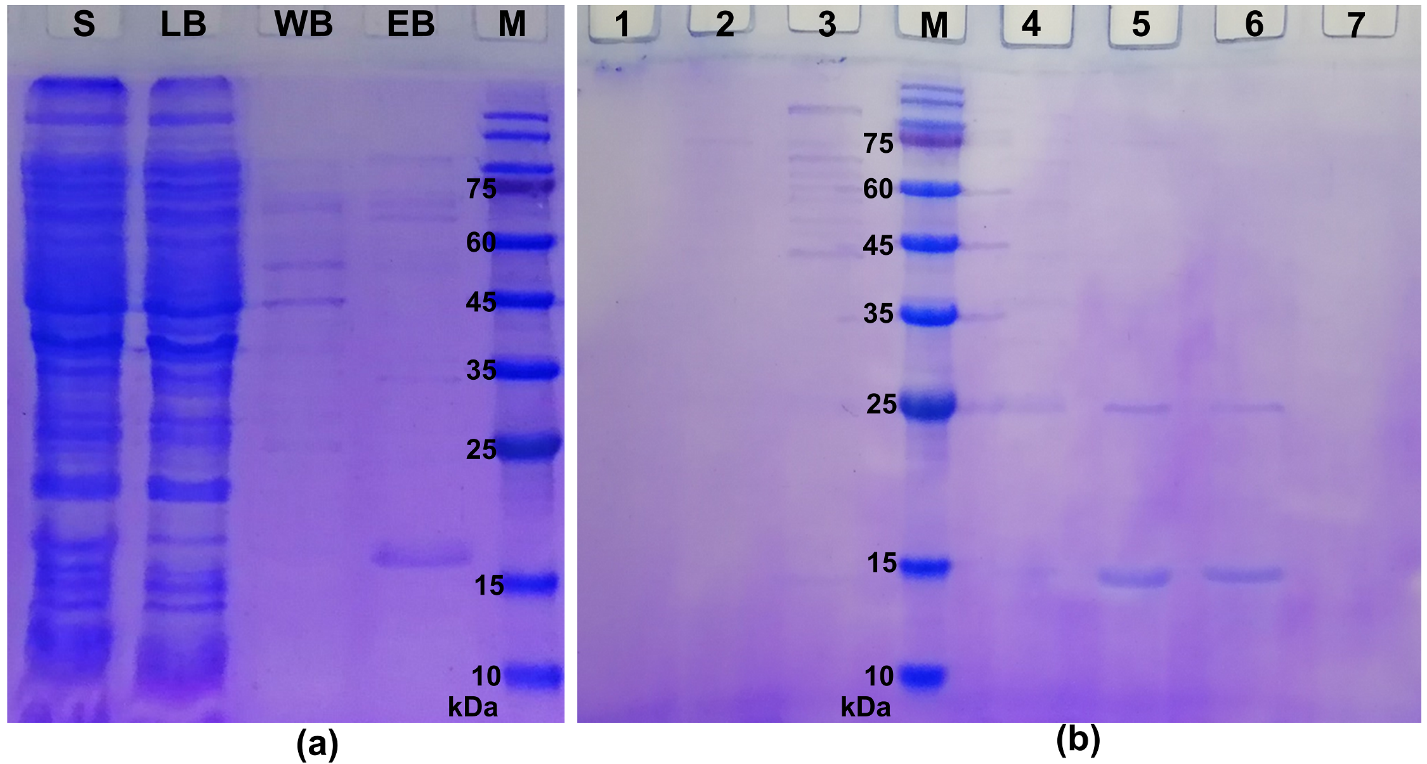


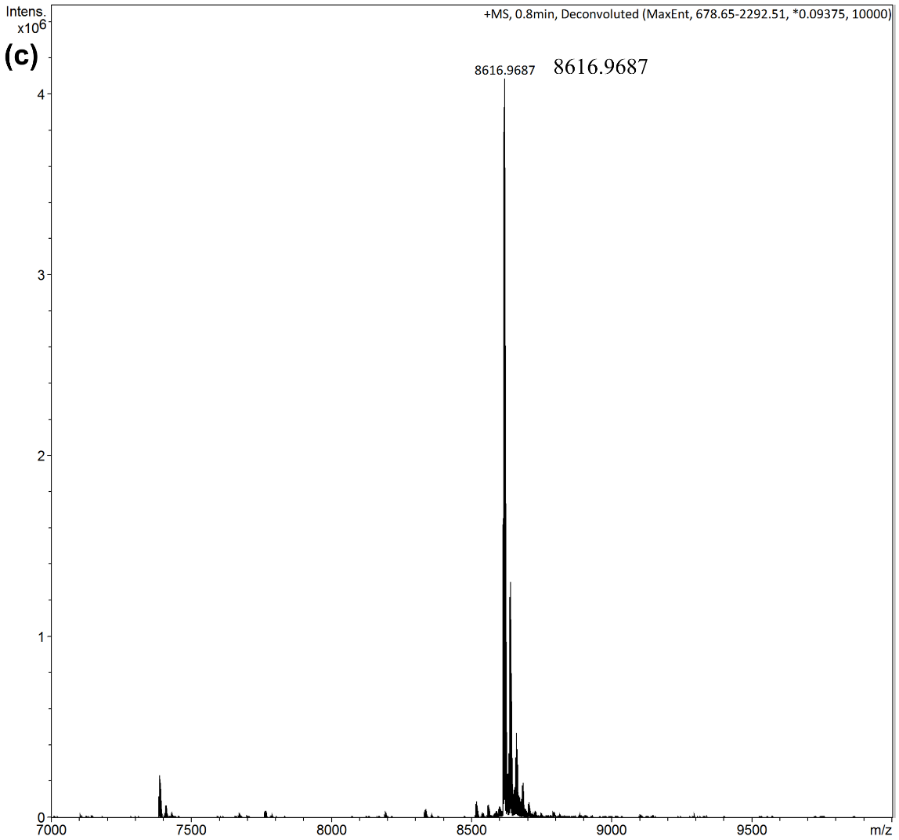


**S4 Fig.** **The p53 (1-73) protein purity and the mass confirmation.** (a) SDS-PAGE demonstrating the various fractions of p53 (1-73) protein collected through the NiNATA Superflow resin column. S represents the crude sample loaded onto the column; LB, WB, and EB indicate the elute fractions collected by employing lysis buffer, wash buffer, and the elution buffer. EB fraction contained the p53-Histidine tag fusion protein (10.5 kDa) which comes above the 15 kDa band before enzyme digestion. Following enzyme digestion with Thrombin, fusion protein after enzyme digestion was loaded onto the Superdex 75 (SEC) column. (b) SDS-PAGE indicating the fractions (1 to 7) collected after enzyme digestion and cleaved p53 (1-73) protein (8.6 kDa) is observed (fraction, 5 and 6) close to the 15 kDa band, though fraction 7 has less protein concentration. (c) Confirmation of the molecular weight of the cleaved p53 (1-73) protein via ESI-MS analysis.
